# A new family of glutamate-gated chloride channels in parasitic sea louse *Caligus rogercresseyi*: A subunit refractory to activation by ivermectin is dominant in heteromeric assemblies

**DOI:** 10.1371/journal.ppat.1011188

**Published:** 2023-03-14

**Authors:** Felipe Tribiños, Patricio Cuevas, Isabel Cornejo, Francisco V. Sepúlveda, L. Pablo Cid

**Affiliations:** 1 Centro de Estudios Científicos (CECs), Valdivia, Chile; 2 Facultad de Ciencias para el Cuidado de la Salud, Universidad San Sebastián, Valdivia, Chile; 3 Facultad de Medicina y Ciencia, Universidad San Sebastián, Valdivia, Chile; Heidelberg University, GERMANY

## Abstract

Sea louse ectoparasitosis is a major threat to fish aquaculture. Avermectins such as ivermectin and emamectin have been effectively used against sea louse infestation, but the emergence of resistance has limited their use. A better understanding of the molecular targets of avermectins is essential to the development of novel treatment strategies or new, more effective drugs. Avermectins are known to act by inhibiting neurotransmission through allosteric activation of glutamate-gated chloride channels (GluCls). We have investigated the GluCl subunit present in *Caligus rogercresseyi*, a sea louse affecting aquaculture in the Southern hemisphere. We identify four new subunits, CrGluCl-B to CrGluCl-E, and characterise them functionally. CrGluCl-A (previously reported as CrGluClα), CrGluCl-B and CrGluCl-C all function as glutamate channel receptors with different sensitivities to the agonist, but in contrast to subunit -A and -C, CrGluCl-B is not activated by ivermectin but is rather antagonised by the drug. CrGluCl-D channel appears active in the absence of any stimulation by glutamate or ivermectin and CrGluCl-E does not exhibit any activity. Notably, the expression of CrGluCl-B with either -A or -C subunits gives rise to receptors unresponsive to ivermectin and showing altered response to glutamate, suggesting that coexpression has led to the preferential formation of heteromers to which the presence of CrGluCl-B confers the property of ivermectin-activation refractoriness. Furthermore, there was evidence for heteromer formation with novel properties only when coexpressing pairs E/C and D/B CrGluCl subtypes. Site-directed mutagenesis shows that three transmembrane domain residues contribute to the lack of activation by ivermectin, most crucially Gln 15’ in M2, with mutation Q15’T (the residue present in ivermectin-activated subunits A and C) conferring ivermectin activation to CrGluCl-B. The differential response to avermectin of these *Caligus rogercresseyi* GluClsubunits, which are highly conserved in the Northern hemisphere sea louse *Lepeophtheirus salmonis*, could have an influence on the response of these parasites to treatment with macrocyclic lactones. They could serve as molecular markers to assess susceptibility to existing treatments and might be useful molecular targets in the search for novel antiparasitic drugs.

## Author summary

Fish is used the world over as a source of protein for a growing population. Fish farm productivity is severely menaced by outbreaks of sea lice, small crustaceans which feed on the skin of fish damaging their muscle and fat, making them unmarketable. Macrocyclic lactone compounds known as avermectins have been used to combat sea louse infestation. Avermectins act by irreversibly activating sea louse glutamate-gated chloride channels, GluCl, disrupting nerve signals leading to paralysis and death. Unfortunately, resistance to avermectins has developed markedly, decreasing their usefulness. To better understand the interaction between avermectins and its target in sea louse we have studied the GluCl subunits in *Caligus rogercresseyi*, the main farmed-fish pest in the Southern Hemisphere. We found that *C*. *rogercresseyi* GluCls form a family of five, named A to E, subunits encoded by separate genes. Of note, subunit B is not activated by avermectins and is able to confer this property to other GluCls when expressed together. We find that *C*. *rogercresseyi* GluCl subunits are highly conserved in the main Northern Hemisphere parasitic sea louse *Lepeophtheirus salmonis*. Together they might serve as molecular markers to assess susceptibility to existing treatments and could become useful molecular targets in the search for novel antiparasitic drugs.

## Introduction

Sea lice are ectoparasite copepods of the Caligidae family (order Siphonostomatoida). These small crustaceans attach to fish and feed on their epidermal tissue and blood and in a fish farming setting lead to morbidity and mortality with extremely high economic impact in the industry. *Caligus rogercresseyi* [1] is the most important parasite in salmonid farming in Chile causing increased costs and decreased productivity with high economic and social impact in the nationally important aquaculture industry [2–4]. Closely related *Lepeophtheirus salmonis* is responsible for high morbidity and mortality in Northern hemisphere salmon and trout aquaculture [5].

Various chemical treatments have been used to counter sea lice infestation of fish farms [2], among them are macrocyclic lactone avermectins emamectin and ivermectin widely used as antiparasitic drugs in human and veterinary medicine [6–8]. The importance of ivermectin has been widespread for more than 35 years, with its discoverers being distinguished with the Nobel Prize in Physiology or Medicine in 2015 [9]. In Chile, emamectin benzoate was administered orally with fish feed to provide long-lasting protection against all forms of attached sea lice, but the success of avermectins as antiparasitic treatment has been threatened by the emergence of resistance that has become a problem worldwide [10–13].

Inhibitory glutamate-gated chloride channels (GluCls) are Cys-loop ligand-gated ion channels (LGICs) made of five subunits whose association can form homomeric or heteromeric receptors [14,15]. Amino acids in transmembrane domain M2 and in the M1-M2 linker determine the anion selectivity of GluCl channels [16]. Glutamate binds at an agonist site at the extracellular domain to open the channel and ivermectin binds at the transmembrane domain and stabilizes an open-pore conformation [17]. GluCls are expressed on neurons and muscle of protostomes where they mediate inhibitory synaptic currents to modulate neuronal and muscle cell excitability [18]. Avermectins interfere with synaptic transmission in arthropods and nematodes through irreversible activation of their GluCls. The ensuing influx of chloride leads to the hyperpolarization of the neuron or muscle which effectively precludes excitability. The interference with essential functions such as feeding and locomotion leading to paralysis and death of the parasite. Ivermectin can activate or potentiate some vertebrate LGICs including chloride channel GABA [19] and Glycine [20] receptors, P2X purinoreceptors [21], and the nicotinic α 7 receptor [22], albeit at a much greater concentration than its effect on invertebrate GluCls. In principle this increases the safety margin of the drug when used on humans or animals, although genetic failure in drug clearance can lead to ivermectin toxicity [23–25].

Possible ways in which drug resistance can arise include a change in the pharmacological target causing the failure of the drug to bind or transduce its binding into the molecular effect, changes in xenobiotic metabolic processes or active transport of the drug from the parasite [26,27], the host or both perhaps by drug-induced differential gene expression [28,29]. Recent transcriptome assays have shown that resistant sea lice have decreased levels of mRNA expression of neuronal acetylcholine or GABA-Cl, but not GluClα, receptors [30,31]. It is not clear how these changes might relate to resistance.

Changes in the expression of the more obviously functionally relevant drug-exporting ABC transporter P-glycoprotein (pgp) have also been explored in sea louse as a possible cause of avermectin resistance. Emamectin exposure has been shown to induce pgp expression in some studies [28,32] but not in others [31,33].

Detailed knowledge of the pharmacological target of ivermectin and emamectin benzoate of *C*. *rogercresseyi* could help to understand the resistance mechanisms and in the design new drugs. In a previous study we cloned a complete transcript of a GluCl from *C rogercresseyi*, studied its function in *Xenopus* oocytes by electrophysiological methods and used molecular modelling better to understand its mode of interaction with avermectins [34]. This subunit, originally termed CrGluClα, is activated by glutamate and irreversibly by ivermectin and emamectin, and mediates chloride currents.

We now identify four new CrGluCl complete transcripts bringing the number of these subunits in *C*. *rogercresseyi* to five. We call the new subunits, that are encoded by separate genes, CrGluCl-B to CrGluCl-E and rename as CrGluCl-A the already identified CrGluCl-α. A survey of their functional properties reveals that all, except for CrGluCl-E, are functional and can be activated by glutamate but vary in their sensitivity to ivermectin. CrGluCl-B is not activated by ivermectin and, remarkably, it confers this refractoriness to ivermectin activation to new receptors arising from coexpression with normally ivermectin-activated CrGluCl-A and CrGluCl-C. We identify transmembrane domain residues that determine CrGluCl-B failure to be activated by ivermectin. It has not escaped our notice that these newly discovered functional differences could have a bearing on the parasite sensitivity to avermectins.

## Results

### Glutamate-gated chloride GluCl channels are represented by five separate transcripts capable of coding for full polypeptides

Mining the Transcriptome Shotgun Assembly (TSA) database at https://ww.ncbi.nlm.nih.gov/ with a “*Caligus rogercresseyi* glutamate-gated chloride channel” query revealed 19 transcript sequences. Three of these (Group A, S1 Fig) correspond to partial fragments of the already identified and characterised subunit [34] that we now rename CrGluCl-A, and one belonged to a glycine receptor and was therefore discarded from further analysis. Four transcripts (Group B, S1 Fig) exhibited overlapping with each other and their assemblage suggested their coding for a full GluCl peptide. The putative protein thus encoded was termed CrGluCl-B. The remaining 11 transcripts were assigned by BLAST analysis against transcripts in the data base for *L*. *salmonis* (https://licebase.org/) and genomic information for *C*. *rogercresseyi* at the https://ww.ncbi.nlm.nih.gov/. With this approach they could be classified in three putative separate subunits that we termed CrGluCl-C, CrGluCl-D and CrGluCl-E (Groups C-E, S1 Fig).

**Fig 1:**
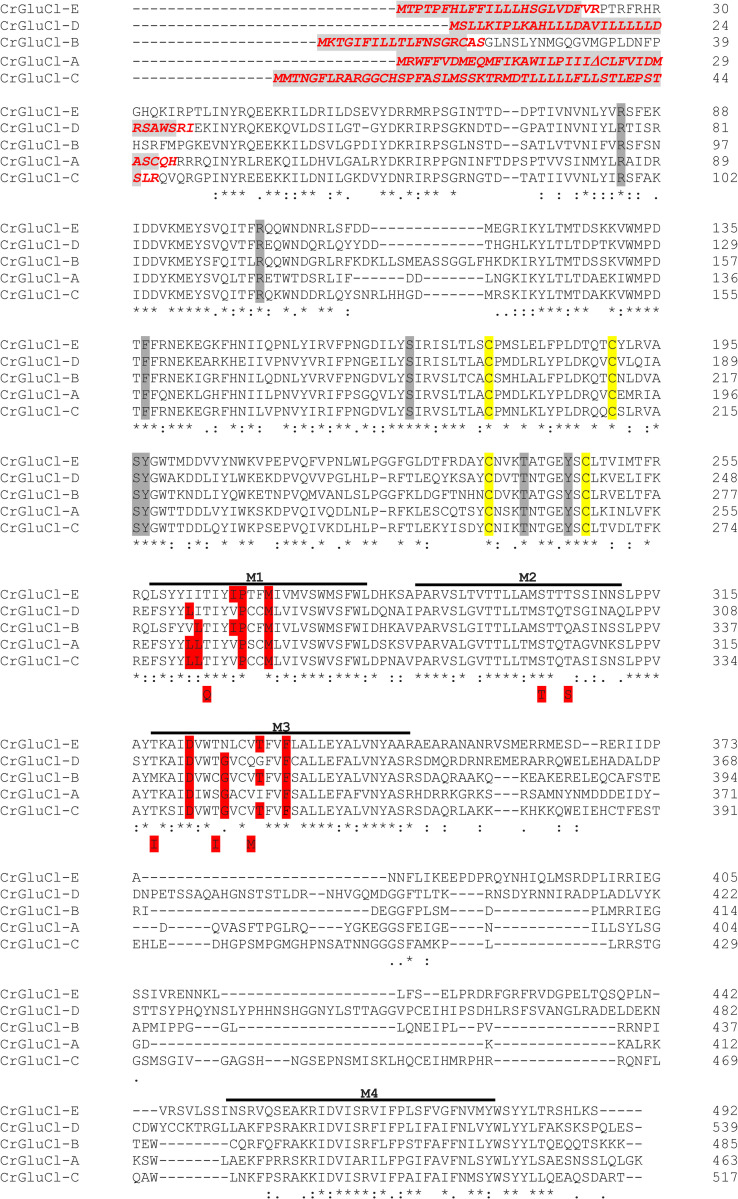
Predicted amino acid sequences for CrGluCl-A, -B, -C, -D and -E. Putative signal peptides (SignalP 4.1 Server) are shown in red italics with the cut portions shaded in gray. Cysteine residues necessary to the formation of C-loops are highlighted in yellow; in grey and in red are residues involved in glutamate and ivermectin binding respectively. The position of the transmembrane domains and binding sites, as well as the residues involved in glutamate and ivermectin binding, are based on the crystal structure of the *C*. *elegans* GluClα [17]. Highlighted in red and below the sequences are amino acids that participate in ivermectin binding in *C*. *elegans* GluClα but are not conserved in the CrGluCl subunits. The sequences for CrGluCl-A, B, C, D, and E are deposited in the GenBank under identifiers KX599189, OP737386, OP737387, OP737388 and OP737389 respectively. Alignments generated using Clustal Omega (https://www.ebi.ac.uk/Tools/msa/clustalo/).

Full cDNAs were generated by RT-PCR from adult *C*. *rogercresseyi* specimens from which full-length putative peptide sequences of CrGluCl-B, CrGluCl-C, CrGluCl-D and CrGluCl-E were obtained. These sequences are shown together with that for CrGluCl-A in the multiple alignment of Fig 1.

The predicted amino acid sequences for CrGluCl-A, -B, -C, -D and -E (Fig 1) all showed the general features characteristic of previously identified glutamate-gated chloride channel subunits. They all have putative signal peptides consistent with a plasma membrane protein expression. A large conserved N-terminal domain exhibited cysteine residues necessary to the formation of pLGIC signature Cys-loops. These were followed by four predicted transmembrane domains (M1-M4) and a shorter C-terminus. Amino acids identified to participate in the binding sites for glutamate and ivermectin [17] are largely conserved in the CrGluCls. Table 1 shows that identities and similarities between the CrGluCls are homogeneously around 50% and 63% respectively. These subunits most likely arise from separate genes.

**Table 1:** Comparison between amino acid sequences of subunits CrGluCl-A to -E. Sequences were analysed across their entire span using the Needleman-Wunsch algorithm (https://blast.ncbi.nlm.nih.gov/Blast.cgi). Positives are matches considered as conservative substitutions.

	Identities (%)	Positives (%)	Gaps (%)
**A *vs*. B**	46	62	11
**A *vs*. C**	51	65	14
**A *vs*. D**	50	62	17
**A *vs*. E**	47	63	12
**B *vs*. C**	54	67	10
**B *vs*. D**	44	57	19
**B *vs*. E**	53	65	13
**C *vs*. D**	52	64	13
**C *vs*. E**	51	65	9
**D *vs*. E**	47	59	14

Sea louse *L*. *salmonis* sequences present in the licebase (https://licebase.org/) and NCBI (https://www.ncbi.nlm. nih.gov/) databases were obtained using the CrGluCls identified here. Indeed, and as seen in S2 Fig, it is possible to identify virtually complete contigs for *L*. *salmonis* GluCls, LsGluCl, homologous to CrGluCl-B and -D, while contigs for putative LsGluCls -A, -C and -E can be generated on the basis of overlapping sequences.

**Fig 2:**
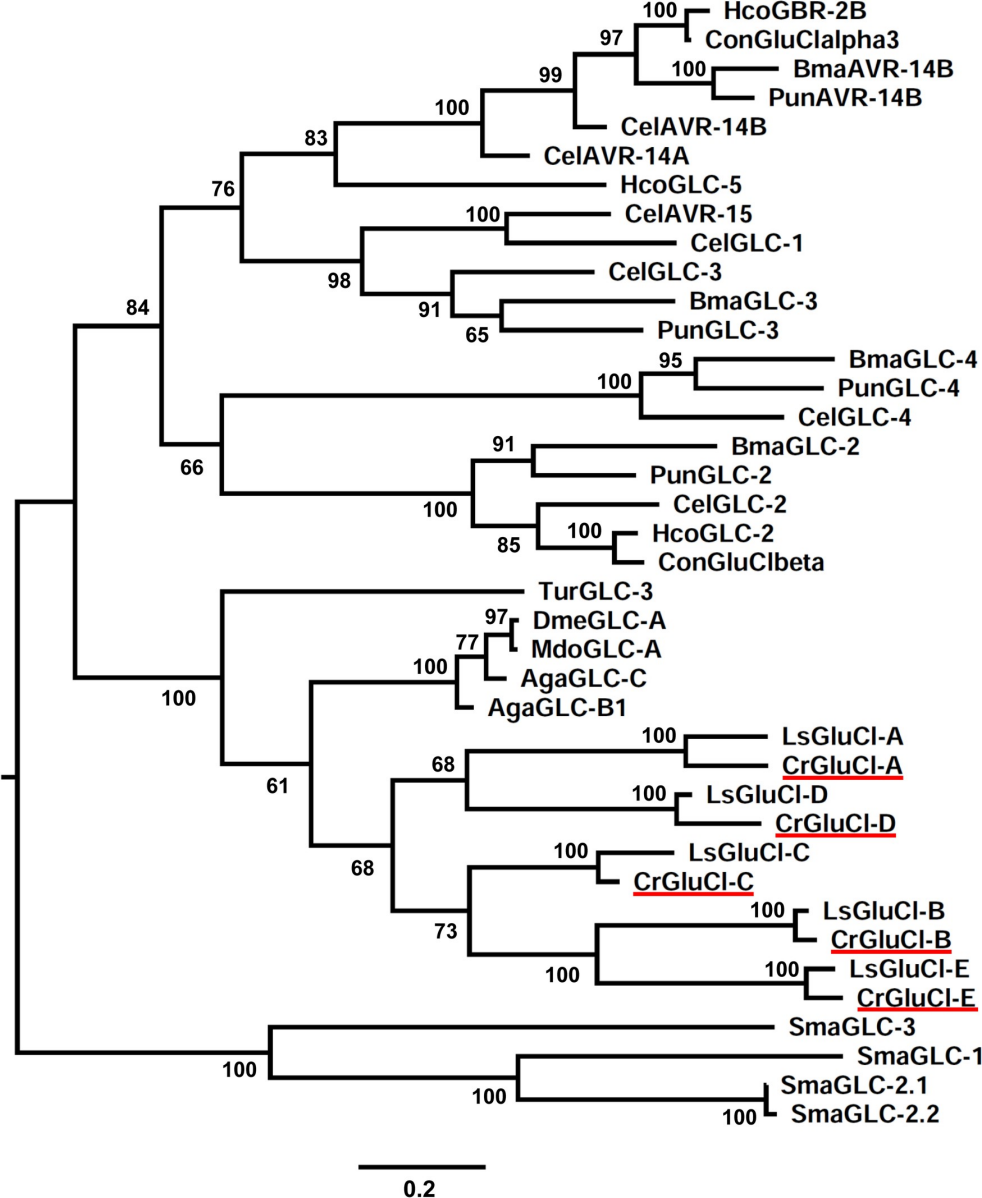
Phylogenetic analysis of CrGluCl subunits. Maximum likelihood tree showing the evolutionary relationship of *C*. *rogercresseyi* GluCl subunits (underlined in red) compared to other arthropods and nematodes CrGluCl counterparts. The percentage of trees in which the associated taxa clustered together is shown next to the branches (100 bootstrap replicates). The tree is drawn to scale, with branch lengths measured in the number of substitutions per site (scale bar). The analysis involved 39 amino acid sequences. The GenBank accession numbers of the amino acid sequences are given in the Methods section. Evolutionary analyses were conducted in MEGA11 [55]. The GluCl subunits of the platyhelminth *Schistosoma mansoni* (SmaGluCl-1, SmaGluCl-2, SmaGluCl-3 and SmaGluCl-4) were used as control since they belong to an independent GluCl clade evolutionarily distinct from arthropod and nematode counterparts [43].

A phylogenetic analysis of these putative *C*. *rogercresseyi* GluCl subunits was made including presently obtained GluCls from *L*. *salmonis*, and GluCls from other arthropods and chosen nematodes for which functional expression has been documented (Fig 2). Receptor subunits from nematodes and arthropods share separate ancestors. Within the arthropod subunits, those belonging to crustaceans *C*. *rogergresseyi* and *L*. *salmonis* made up a clade separate from those belonging to insects and the arachnid *T*. *urticae*. Each of the *C*. *rogercresseyi* subunits is orthologous to the *L*. *salmonis* gene products reported in S2 Fig This conclusion is supported by branch reliability estimates using bootstrapping.

### Functional expression of *Caligus rogercresseyi* putative glutamate-gated chloride channels

Evaluation of the functional properties of *C*. *rogercresseyi* GluCl subunits was carried out after expression in *Xenopus laevis* oocytes by microinjection of cRNA derived from cDNA of variants CrGluCl-A, -B, -C, -D and -E [34].

### CrGluCl-B

**Fig 3:**
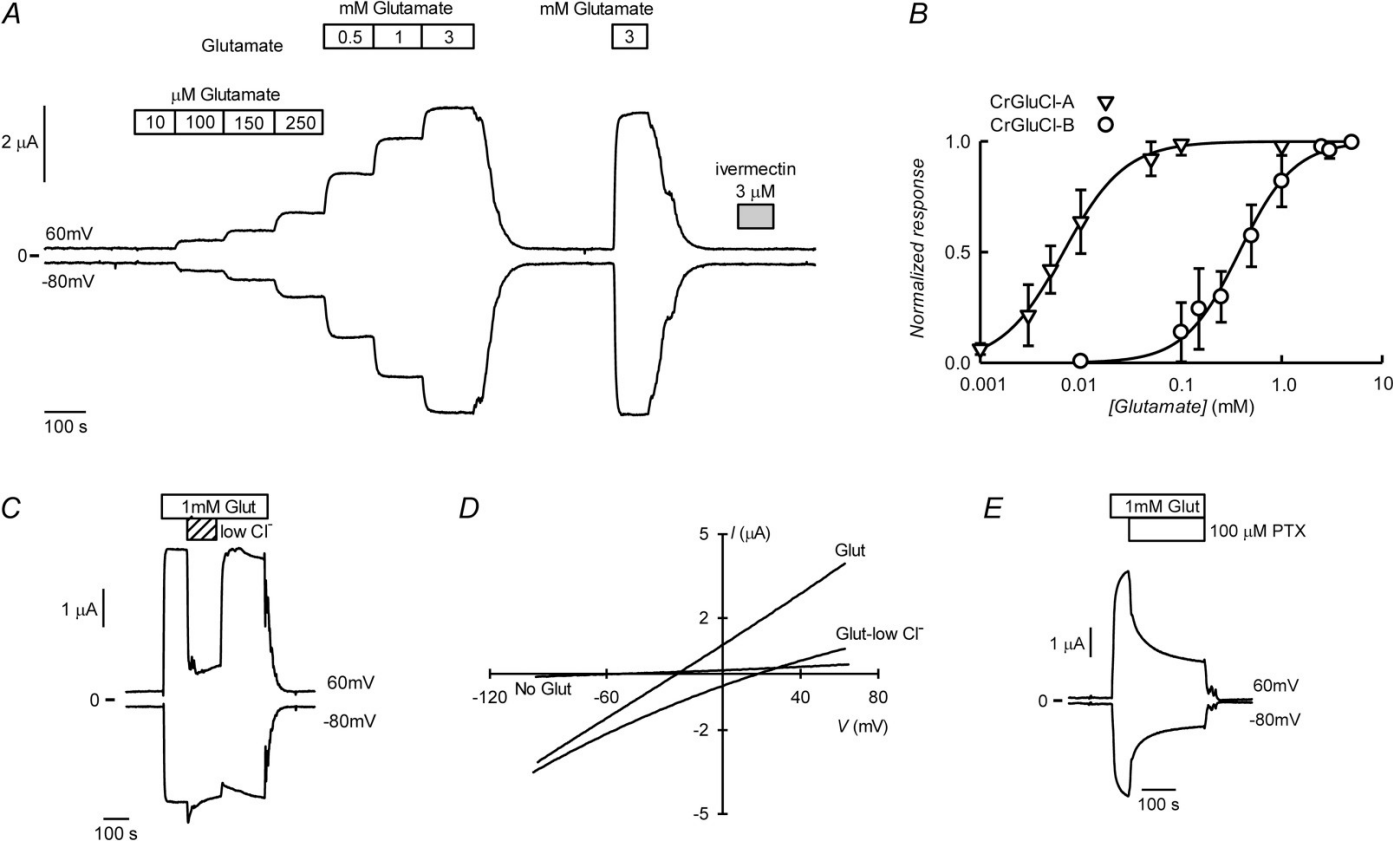
Glutamate-activated currents of CrGluCl-B cRNA-injected *Xenopus* oocytes. A. Current traces obtained at the indicated membrane potentials during bath application of glutamate at concentrations from 10 μM to 3 mM, or 3 μM ivermectin during the times shown in the boxes. B. Plot summarising results of the concentration-response relationship of the glutamate-sensitive currents mediated by CrGluCl-B. The responses were normalised to the maximal effect evoked by glutamate. The lines are Hill equations fitted to the data sets which are means ± SD with n = 16 for CrGluCl-B. Data for CrGluCl-A (n = 5) are from experiments performed contemporarily. C. Chloride -dependence of 1 mM glutamate-evoked current. D. Current voltage relations taken from voltage-ramps applied during the experiment shown in panel C. The graph shows a current-voltage relation before addition of glutamate (No Glut), in the presence of glutamate (Glut) and in low chloride extracellular solution. E. Effect of 100 μM PTX on glutamate-activated currents mediated by CrGluCl-B.

CrGluCl-A (formerly called CrGluClα) has been previously expressed in *Xenopus* oocytes and showed in electrophysiological assays to be activated by glutamate to promote chloride currents that could be blocked by the ligand-gated anion channel inhibitor picrotoxin (PTX). Avermectins ivermectin and emamectin activate CrGluCl-A irreversibly with EC_50_ values of around 200 nM [34]. Similar assays performed now using CrGluCl-B also show that this subunit behaves as a glutamate-gated chloride channel (Fig 3). The graph shows current traces obtained using two-electrode voltage-clamp of an oocyte previously injected with CrGluCl-B cRNA. Outward and inward currents were recorded respectively at 60 and -80 mV, which correspond to chloride flowing into and out of the oocyte respectively (Fig 3A). Superfusion with increasing concentrations of L-glutamate led to a graded increase in current. Much as reported for CrGluCl-A [34], there is no desensitization of the response of CrGluCl-B to glutamate. In contrast with the glutamate response, ivermectin was without effect on the currents revealing a complete refractoriness to activation of CrGluCl-B by the antiparasitic drug. Fig 3B shows the dose dependence of the glutamate effect on CrGluCl-B measured at 60 mV. The average relationship, reported as circles in the figure, can be described by a Hill equation. Separate Hill equation fits to these experiments gave an EC_50_ of 388 ± 203 μM and n_H_ of 1.7 ± 0.1 (Mean ± SD, n = 16). The result of similar experiments using CrGluCl-A are also shown as downward pointing triangles. These summarise individual Hill equation fits giving an EC_50_ of 7.2 ± 2.7 μM and n_H_ of 1.65 ± 0.63 (Mean ± SD, n = 5). Previously published values for glutamate CrGluCl-A EC_50_ and n_H_ were 6.9 μM and 1.33 respectively [34].

Although all CrGluCl channels have roughly similar selectivity filters, some divergences occur at M2 that might alter ion selection. Divergences are present in CrGluCl-A at positions 2´, 17´and 18´; CrGluCl-B at positions 5´, 10´, 14´and 15´; CrGluCl-C at position 19´; CrGluCl-D at positions 16´and 17´; CrGluCl-E at positions 4´, 10´, 14´and 16´). In addition, changes of the structure elsewhere from M2 might also affect the conformation of the selectivity filter and its interaction with the permeant ion. Picrotoxin, on the other hand, is a selectivity filter blocker and these divergences in M2 might also affect its potency. We have therefore checked both selectivity and picrotoxin effect for the different CrGluCls.

Fig 3C shows that the glutamate-dependent current was carried by chloride, as partial extracellular replacement Cl^-^ with a more impermeant anion sharply reduced outward current (Cl^-^ influx). This is corroborated by data from the current-voltage (IV) relation taken during glutamate application under normal and low extracellular chloride (Fig 3D). Data are taken from voltage-ramps applied during the experiment in panel C. The graph shows a current-voltage relation before addition of glutamate (No Glut). This small current reversed at a potential, E_rev_, of -50 mV. In the presence of glutamate (Glut), an increased current is seen to occur at all voltages tested with the current vs. voltage relationship nearly linear. In low Cl^-^ extracellular solution E_rev_ became depolarized and the IV relation appeared slightly inwardly rectified, as expected for a current carried by chloride. On average E_rev_ for the glutamate-activated current shifted from -25 ± 6.2, close to the chloride equilibrium potential of *Xenopus* oocytes [30], to 8 ± 8.2 mV in low chloride, giving a calculated P_Gluconate_/P_Cl_ value of 0.2 ± 0.06 (means ± SD, n = 6). Fig 3E shows that 100 μM PTX inhibits the currents elicited by 1 mM glutamate. Average inhibition was 76 ± 9% (mean ± SD, n = 4).

**Fig 4:**
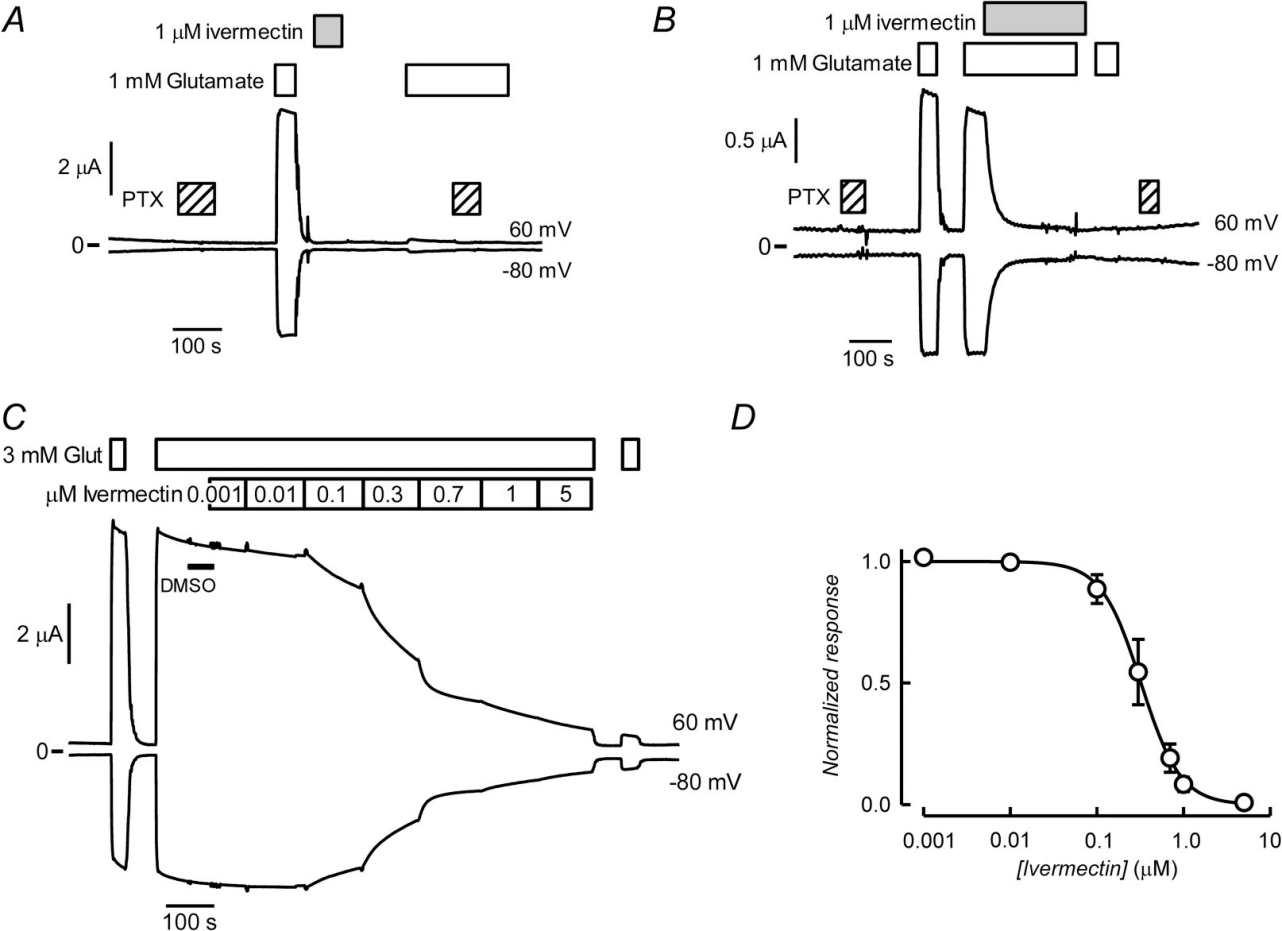
Ivermectin antagonises the effect of glutamate on CrGluCl-B. A and B. Effect of ivermectin on the response of CrGluCl-B to glutamate. Current traces were obtained at the indicated membrane potentials during bath application of 1 mM glutamate as affected by ivermectin application at 1 μM during the times shown in the boxes. The current evoked by glutamate was 4.67 ± 1.17 μA before and 1.70 ± 1.69 after ivermectin treatment (means ± SD, n = 7). PTX shows addition of 100 μM PTX. Black bar labelled DMSO indicates addition of 0.05% dimethyl sulfoxide, the highest concentration reached for the ivermectin solvent. C. Shows the effect of increasing ivermectin concentrations on CrGluCl-B current elicited by 3 mM glutamate addition. In D the extent of antagonism is illustrated in the ivermectin inhibitory concentration-response curve. The responses were normalised to the maximal effect evoked by glutamate. The line is a Hill equations fitted to the data sets which are means ± SD (n = 7).

Ivermectin and other compounds of the avermectin family of macrocyclic lactones are potent and irreversible activators of CrGluCl-A and some other ionotropic invertebrate receptors. However, as shown above ivermectin fails to increase the activity of CrGluCl-B. Although avermectins do not produce the expected activation of CrGluCl-B, they act as antagonists of this subunit as illustrated in Fig 4A and 4B. In A a first application of the agonist glutamate elicits a robust and reversible activation of CrGluCl-B while subsequently added ivermectin is without effect. A second, post-ivermectin stimulation with glutamate however fails to elicit a response. There is no attenuation of the glutamate response upon a second addition of the agonist without intervening treatment with ivermectin, but the drug is able to abolish activity in the continued presence of glutamate (Fig 4B). This antagonistic effect is also observed when using emamectin, a different avermectin (S3A and S3B Fig). Increasing concentrations of ivermectin were used to gauge the potency of its antagonism of the glutamate-elicited current of CrGluCl-B. Fig 4C shows CrGluCl-B currents recorded in the continuous presence of 3 mM glutamate as affected by ivermectin that elicits a graded inhibition. This is quantified in the dose response relation in Fig 4D. The data can be described by a Hill equation. Separate Hill equation fits to individual measurements gave an IC_50_ of 342 ± 114 nM and n_H_ of -2.2 ± 0.5 (mean ± SD, n = 7).

### CrGluCl-C

**Fig 5:**
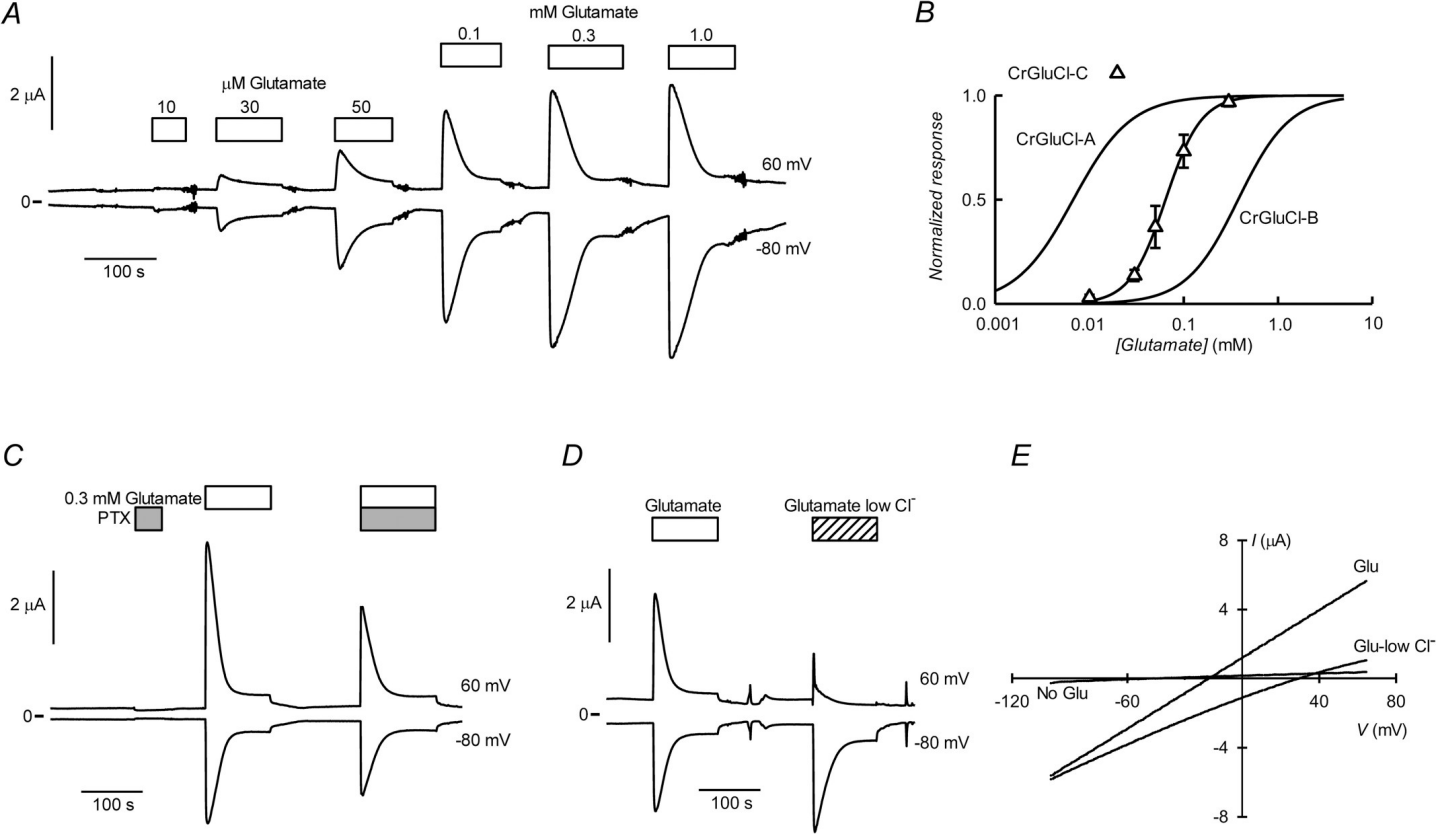
Glutamate-evoked currents in oocytes injected with CrGluCl-C cRNA. A. Current traces obtained at the indicated membrane potentials during bath application of increasing glutamate concentrations as indicated by the boxes. B. Plot summarising results of the concentration-response relationship of the glutamate-sensitive current mediated by CrGluCl-C (means ± SD, n = 6). C. Effect of 100 μM PTX on the glutamate-dependent current of an oocyte expressing CrGluCl-C. D. Current traces obtained at the indicated membrane potentials during bath application of 0.3 mM glutamate with normal and low extracellular chloride concentration as indicated by the boxes. E. The respective current-voltage relations taken from voltage-ramps applied during experiment in D are shown.

Fig 5 shows the results of the functional assay of CrGluCl-C. Contrasting with the results with variants A and B, CrGluCl-C activation by glutamate results in a transient response (Fig 5A) at all concentrations tested. The concentration dependence of the peak response to glutamate is given in Fig 5B where it is compared with those of the A and B subunits. The average relationship (mean ± SD, n = 6), reported by upright triangles in the graph, can be described by a Hill equation. Separate fits to these experiments gave an EC_50_ of 67 ± 25 μM and n_H_ of 2.5 ± 0.56 (Mean ± SD, n = 6).

**Fig 6:**
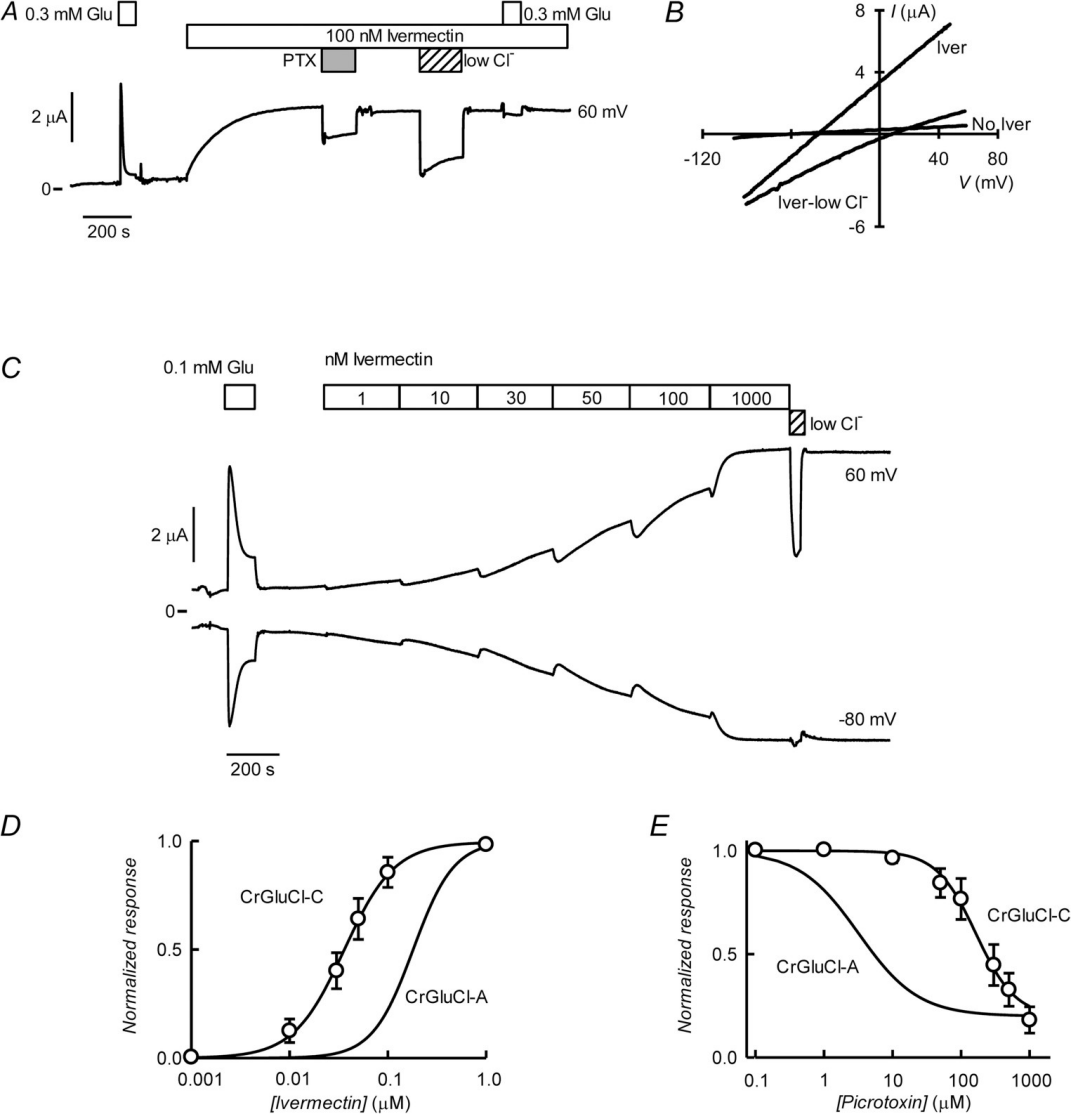
Irreversible activation of CrGluCl-C by ivermectin. A. Current trace obtained at 60 mV during bath application of 300 μM glutamate and then ivermectin at 100 nM during the times shown in the boxes. The effect of PTX addition at 100 μM and the reduction in extracellular chloride concentration are also shown. B. Current voltage relations taken from voltage-ramps applied during the experiment in A before and after ivermectin addition as well as after superfusion with the low chloride solution. C. Effect of increasing concentrations of ivermectin on CrGluCl-C and the effect of partial external chloride removal. D. Effect of increasing concentrations of ivermectin on CrGluCl-C reported as average dose dependence of the response. Data (mean ± SD, n = 7) are normalized to the maximal effect of ivermectin. The line is a fit of the Hill equation to the values and it is compared to that measured for CrGluCl-A previously [34]. E. Effect of increasing concentrations of picrotoxin on CrGluCl-C current elicited by 1 μM ivermectin. Data (means ± SD, n = 9) are normalized to ivermectin-dependent current value before PTX addition. The line is a fit of the Hill equation to the values and it is compared to that measured for CrGluCl-A previously [34].

CrGluCl-C was only partially inhibited by PTX at 100 μM (23 ± 10% inhibition, mean ± SD, n = 9, Fig 6E). The same concentration of PTX elicits a 78 ± 2% inhibition (mean ± SD, n = 4) of CrGluCl-A (see Fig 6E for a dose-response curve taken from reference 34) and a 76 ± 9% inhibition of CrGluCl-B (mean ± SD, n = 4, see Fig 3E). Anion replacement experiments (Fig 5D and 5E) show that the current through CrGluCl-C is carried by chloride ions. Glutamate-activated outward current shown in Fig 5D was markedly decreased in a low chloride solution. Current voltage relations taken from voltage-ramps applied during this experiment are shown in Fig 5E. There was little rectification for the glutamate-induced current that has an E_rev_ at ~-32 mV. Reduction in extracellular chloride shifts E_rev_ in a depolarized direction and the IV relation acquires some inward rectification, consistent with anion permeation. In separate experiments the observed E_rev_ shift was from -25 ± 6.1 to 8 ± 8.2 mV giving a calculated P_Gluconate_/P_Cl_ value of 0.1 ± 0.09. Spontaneous membrane potential in the absence of agonist was -51 ± 11 (all data means ± SD, n = 5).

CrGluCl-C is sensitive to ivermectin as seen in Fig 6A that shows a recording of CrGluCl-C current at 60 mV and where addition of 0.3 mM glutamate produced the expected transient increase in outward current. After glutamate removal, ivermectin at 100 nM elicited a stable increase in outward current that could be partially inhibited by PTX at 100 μM. That the current is carried by chloride is shown by its decrease by partial removal of the anion from the extracellular solution. Current voltage relations taken from voltage-ramps applied during this experiment are shown in Fig 6B. These gave similar results as for glutamate-activated currents, with little rectification for the ivermectin-induced current which is shifted in the depolarized direction from IV taken before addition. A further shift is seen after low chloride solution perfusion. Respective E_rev_ values were -55, -41 and 10 mV. In the presence of ivermectin the membrane potential shifted from -25 ± 8.9 mV in normal chloride concentration to 18 ± 11 mV in the low chloride solution, giving a calculated P_Gluconate_/P_Cl_ value of 0.13 ± 0.08 (means ± SD, n = 5).

Ivermectin causes graded activation in anion current with increasing concentration and the effect of the drug is irreversible (Fig 6C). The circles in the plot in Fig 6D show the average response of CrGluCl-C to ivermectin and a Hill equation fit to the data. Separate fits to data yielded an EC_50_ value of 31.1 ± 14.4 nM and n_H_ of 2.1 ± 0.67 (means ± SD, n = 7). These compare with values of 181 nM for EC_50_ and n_H_ of 2.1 previously reported for the CrGluCl-A subunit [34]. Maximal current reached at saturating ivermectin concentration was 3.7 ± 1.3 μA (n = 10), which compares with 3.5 ± 1.7 μA for the maximal current elicited by glutamate.

As seen above the sensitivity of CrGluCl-C to PTX appears lower than those of subunits A and B. This was explored further in the PTX inhibitory dose-response in Fig 6E. PTX inhibited ivermectin-induced CrGluCl-C current with an IC_50_ of 275±96 μM (n = 7) and n_H_ -1.2±0.22 (n = 7). PTX potency for CrGluCl inhibition is therefore two orders of magnitude lower than for the A subunit measured previously at IC_50_ of 3.2 μM ([34], fit also shown in the graph for comparison).

### CrGluCl-D

**Fig 7:**
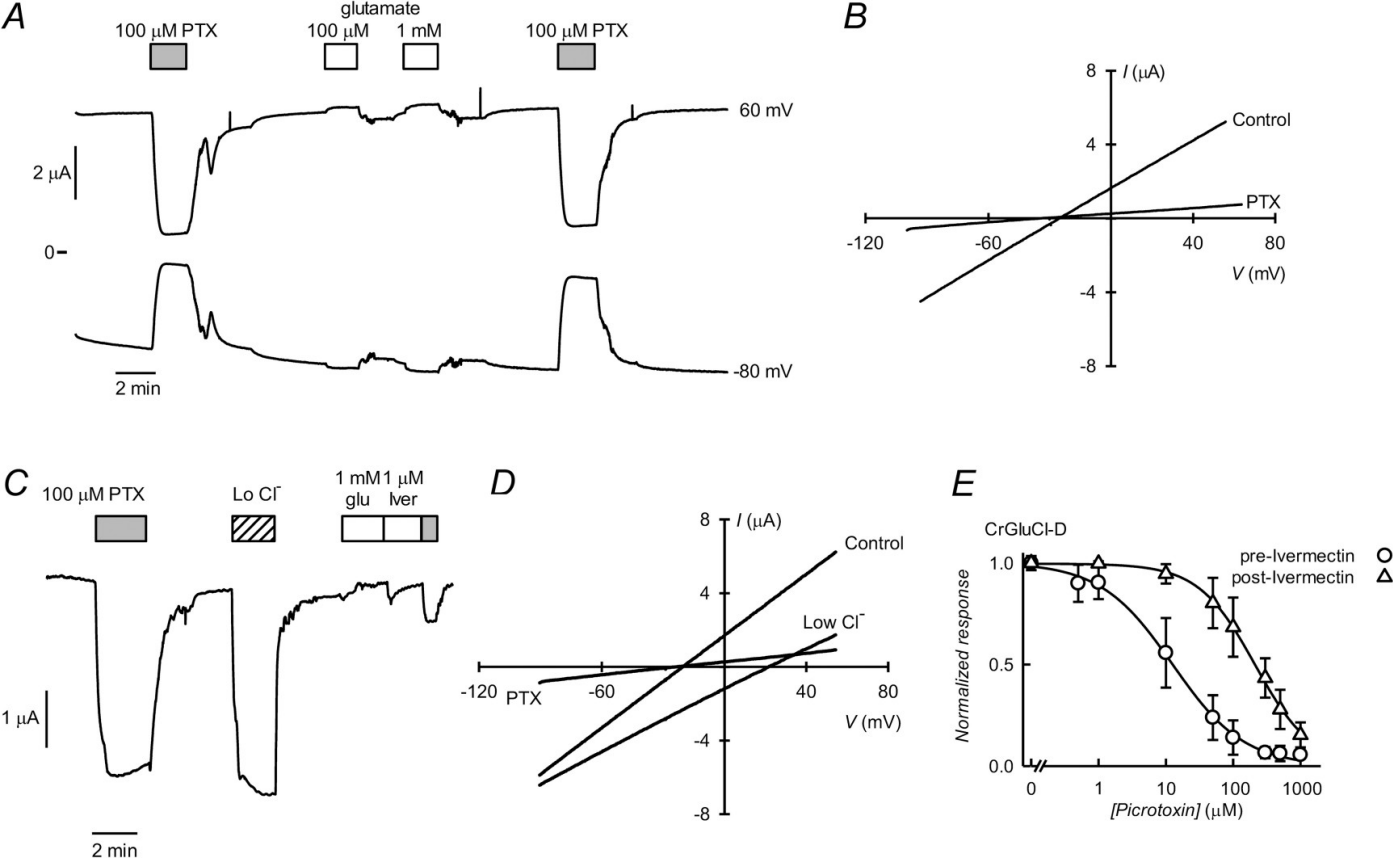
Spontaneously active currents are present in oocytes injected with CrGluCl-D cRNA. A. Currents are measured at the indicated voltages and PTX or glutamate additions are as indicated. Large inward and outward currents were markedly inhibited in a reversible way by PTX. Addition of 100 μM and then 1 mM glutamate produced only a very slight effect B. IV currents taken from A before or after inhibition with 100 μM PTX. C. Traces taken at 60 mV show current chloride dependence and the lack of effect of ivermectin. PTX effect is decreased by previous ivermectin treatment. D. IV curves correspond to trace in C and represent initial control spontaneous current with no addition and in the presence of PTX or low extracellular chloride. E. Effect of increasing concentrations of PTX on currents taken from oocytes expressing CrGluCl-D either without any pre-treatment (pre-ivermectin) or after a ~2 min exposure to 1μM ivermectin (post-ivermectin). Results are means ± SD, n = 11 and 12 respectively.

Expression of subunit CrGluCl-D gives rise to large spontaneous currents (Fig 7A) that amounted to 6.4 ± 0.76 μA (mean ± SD, n = 12) at 60 mV and could be blocked by the ligand-gated anion channel inhibitor PTX. E_rev_ of the spontaneous current was -25 mV (control in 7B), close to E_Cl_. Fig 7C shows that spontaneous outward current is markedly decreased upon lowering extracellular chloride and that although glutamate or ivermectin lack a direct activating effect they modulate PTX-sensitivity of the spontaneous current (Fig 7C). Fig 7D shows IV curves taken from trace in C, and represent initial control spontaneous current with no addition and in the presence of PTX or low extracellular Cl^-^. Partial removal of extracellular chloride displaced E_rev_ from -21 ± 10.2 to 14.5 ± 14.8 mV, giving a P_Gluconate_/P_Cl_ permeability ratio of 0.21 ± 0.12 (means ± SD, n = 4). The sensitivity to PTX of CrGluCl-D was explored both in naïve oocytes expressing CrGluCl-D or in those exposed previously to 1 μM ivermectin. Fig 7E shows the respective dose response data. The IC_50_ for PTX inhibition before ivermectin treatment was 16±8.8 μM while that after ivermectin increased to 221±100 μM (means ± SD, n = 11 and 12 respectively). The spontaneous membrane potential near E_Cl_ of CrGluCl-D-expressing oocytes, the inhibition of their spontaneous currents by PTX, and the changed sensitivity to PTX induced by the GluCl agonist ivermectin treatment, all support the hypothesis that the observed activity corresponds to a permanently active GluCl receptor.

### CrGluCl-E

Functional assay of CrGluCl-E gave low spontaneous currents and failed to show any response to glutamate up to 1mM or to 1μM ivermectin. We presume that CrGluCl-E is an inactive form of glutamate receptor or that the protein fails to reach the oocyte plasma membrane (but see below).

### Functional properties of coexpressed CrGluCl subunits

Invertebrate glutamate receptors belong to the Cys loop ligand-gated ion channel (LGIC) group of proteins that can assemble as homopentamers or heteromers of different stoichiometries. Most of the GluCl subunits successfully expressed *in vitro* have been homomeric [18] but there are instances of heteromeric assembly in coexpression experiments *in vitro* that give rise to activities with novel properties [35–38]. The subunit composition of native GluCls is not known in species with genes coding for multiple subunits. We undertook to explore whether the different subunits of *C*. *rogercresseyi* GluCl identified here might form heteromers with properties distinct from those of the individual subunits when coinjected in *Xenopus* oocytes.

### CrGluCl-A and CrGluCl-B coexpressions

**Fig 8:**
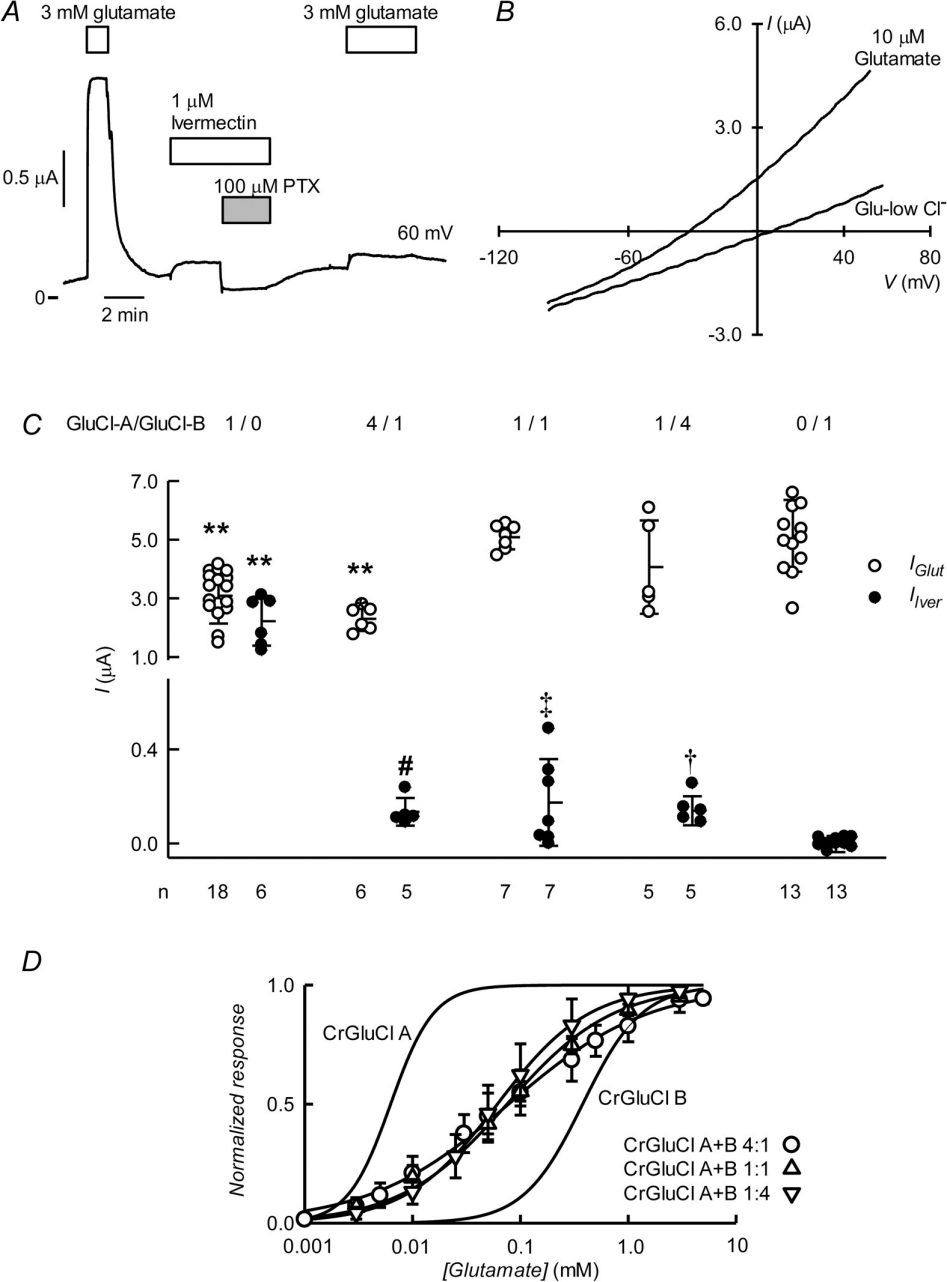
Functional properties of coexpressed CrGluCl-A and CrGluCl-B subunits in *Xenopus* oocytes. A. Currents elicited by 3 mM glutamate or 1 μM ivermectin in an oocyte expressing subunits A and B after microinjection of 16 ng cRNA of CrGluCl-A and 4 ng cRNA of CrGluCl-B. Recording taken at 60 mV. B. IV relations corresponding to control before any addition or during the glutamate (first addition) or ivermectin challenges. C. Summary of currents elicited by glutamate or ivermectin in oocytes expressing CrGluCl-A or CrGluCl-B subunits on their own or at the various proportions indicated. Total cRNA injected was always 20 ng. Data points report individual measurements with means ± SDs also shown, n indicated under the abscissa. Glutamate or ivermectin responses significantly different from those with CrGluCl-B alone (** P<0.001) as analysed by ANOVA with Bonferroni correction. The same test gave no significance for the differences in ivermectin response between any of the CrGluCl-A:CrGluCl-B mixed expressions experiments and that for CrGluCl-B alone. One sample t-test results to assess a significant difference from zero were # = 0.003, ‡ = 0.023 and † = 0.004. D. Dose-response relationship of the glutamate-sensitive currents of CrGluCl-A:CrGluCl-B mixed expression experiments. Data are normalized (means ± SD, n **=** 7, 7 and 5 for the 4:1, 1:1, and 1:4 mixtures respectively) to the maximal effect of. Lines are Hill equation fits. Those for the subunits expressed separately are taken from Fig 3 and shown for comparison.

Coinjection of cRNA for subunits A and B yields activities with characteristics at variance with those of either of the subunits expressed separately. Fig 8A shows a recording of the currents arising after microinjecting *Xenopus* oocytes with a 4:1 CrGluCl-A:CrGluCl-B mixture of cRNAs. These oocytes responded to glutamate with prompt development of robust currents similar in kinetics to those elicited after expression of the separate subunits. There was only a small response to ivermectin and further glutamate addition evoked a little increase in current, reminiscent to what is seen when expressing CrGluCl-B on its own. The IV relationship of the current induced by glutamate was ohmic and had an E_rev_ of -22 mV, a shift from the -52 mV E_rev_ of the preaddition, control IV (Fig 8B). Separate analysis of IV relations of glutamate-activated currents in oocytes expressing a 4:1 CrGluCl-A:CrGluCl-B mixture after partial chloride replacement gave a P_Gluconate_/P_Cl_ permeability ratio of 0.21 ± 0.12 (mean ± SD, n = 3).

Similar results were obtained after expression of 1:1 and 1:4 CrGluCl-A:CrGluCl-B mixtures of cRNAs. A summary of the responses to 3 mM glutamate and 1 μM ivermectin of the three A:B mixtures tested is given in Fig 8C together with the responses of the oocytes injected singly with either CrGluCl-A or CrGluCl-B. All three mixtures presented robust responses to glutamate but those to ivermectin were quite small (notice axis brake and change of scale in the ordinate of Fig 8C). Compared to the glutamate response of CrGluCl-B those of CrGluCl-A and the 4:1 mixed expression are significantly smaller (P<0.001 by ANOVA analysis). The response to ivermectin of all, 4:1, 1:1 and 1:4 mixed A:B-expresing oocytes, did not differ significantly from the (lack of) response to ivermectin of CrGluCl-B, but all three though small, were nevertheless different from zero (P = 0.003, 0.023 and 0.004 respectively as analysed in one sample t-tests).

The EC_50_ values of CrGluCl-A and -B differ significantly. That for CrGluCl-A, at 7 μM, is amongst the lowest reported. Those for helminths and insects CeGluClβ, DmGluClα, HcGluClα and AgGluCl are 380, 23, 28 and 30 μM respectively [36,39,40]. The CrGluCl-B EC_50_ on the other hand is much higher at around 400 μM. Mixed expression of CrGluCl-A and CrGluCl-B glutamate dose-response (shown in Fig 8D together with the curves for CrGluCl-A and CrGluCl-B for comparison) give an intermediate EC_50_ for glutamate that was rather independent of the proportions of the mixtures. EC_50_ and n_H_ values for 4:1, 1:1 and 1:4 mixed A:B-expresing oocytes were respectively 89 ± 47 μM and 0.68 ± 0.10 (n = 7); 82 ± 35 and 0.88 ± 0.19 (n = 7); and 72 ± 47 μM and 1.08 ± 0.16 (n = 5). Values are derived from fits to the individual experiments and are averages ± SDs.

### CrGluCl-C in coexpression with CrGluCl-B or CrGluCl-A

**Fig 9:**
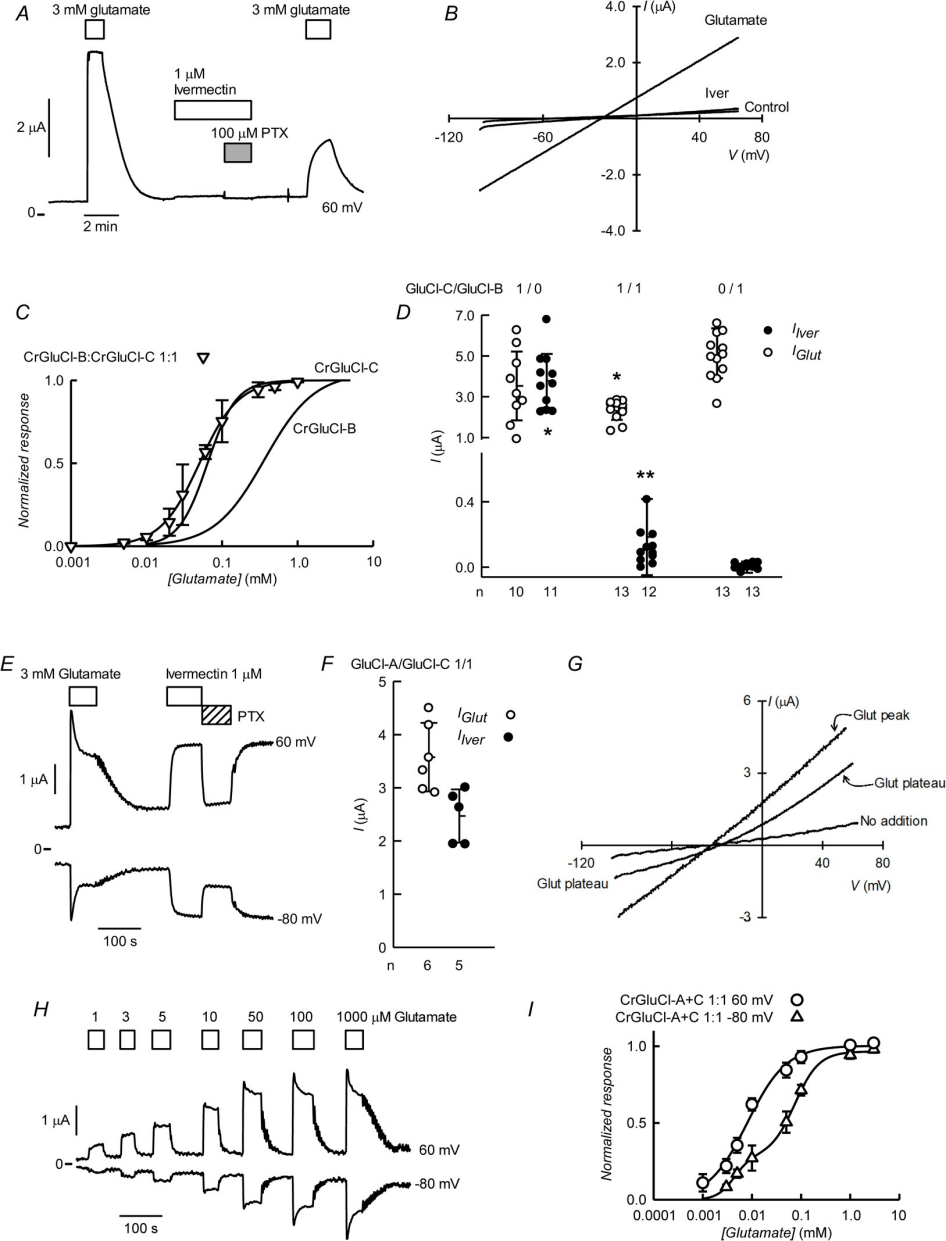
Functional properties of CrGluCl-C with CrGluCl-B, and CrGluCl-C with CrGluCl-A subunits after coinjection into *Xenopus* oocytes. A. Currents elicited by 3 mM glutamate or 1 μM ivermectin in an oocyte expressing subunits C and B after microinjection of 10ng cRNA for each CrGluCl-C and CrGluCl-B. Recording taken at 60 mV. B. IV relations corresponding to control before any addition or during the glutamate (first addition) or ivermectin challenges. C. Dose-response relationship of the glutamate-sensitive currents of CrGluCl-C:CrGluCl-B mixed expression experiments. Data are normalized (mean ± SD, n = 13) to the maximal effect of glutamate. The line is a Hill equation fit. Those for subunits CrGluCl-B and CrGluCl-C expressed separately are taken from Figs 3B and 5B and are shown for comparison. D. Summary of currents elicited by glutamate or ivermectin in oocytes expressing CrGluCl-C or CrGluCl-B subunits on their own, or together in a 1:1 proportion. Total cRNA injected was always 20 ng. Data points report individual measurements with means ± SDs also shown, n indicated under the abscissa. Glutamate or ivermectin responses significantly different from those with CrGluCl-B alone are * P<0.05 as analysed by Kruskal-Wallis one way ANOVA. ** P<0.001 indicates value different from zero in a one-sample t-test. E. Currents elicited by 3 mM glutamate or 1 μM ivermectin in an oocyte expressing subunits C and A after microinjection of 10ng cRNA for each CrGluCl-C and CrGluCl-A. Recording taken at 60 mV and -80 mV. F. Summary of currents measured at 60 mV elicited by glutamate or ivermectin in oocytes expressing CrGluCl-C and CrGluCl-A in a 1:1 proportion. Total cRNA injected was always 20 ng. Data points report individual measurements with means ± SDs also shown, n indicated under the abscissa. G. IV relations corresponding to control before any addition or during the glutamate challenge taken at the peak of the response or at the plateau phase. H. Effect of increasing glutamate concentrations on currents of oocytes coinjected with CrGluCl-C:CrGluCl-A cRNAs at the indicated membrane potentials. I. Average dose-response relationship of the glutamate-sensitive currents of the 1:1 CrGluCl-C:CrGluCl-A coinjected oocytes taken at the peak response at either 60 or -80 mV. Data are normalized (mean ± SD, n = 4) to the maximal effects of glutamate. The lines are Hill equation fits.

Fig 9A shows a current recording from an oocyte injected with a CrGluCl-C:CrGluCl-B 1:1 mixture. Similarly to the CrGluCl-A:CrGluCl-B coexpression, the 1:1 mixture of CrGluCl-C and CrGluCl-B gave rise to a functional receptor with novel properties. Indeed, 3 mM glutamate elicited robust current that, remarkably, lacked the marked desensitization seen with subunit C alone (Fig 5). In addition, 1 μM ivermectin failed to elicit a significant response. As for CrGluCl-B alone or in coexpression with subunit A, the glutamate response was persistently antagonized by previous ivermectin treatment. The IV curve of the glutamate-dependent current was linear and reverted at -22 mV compared with a resting E_rev_ of -52 mV (Fig 9B). The ivermectin-induced IV relation did not differ markedly from the control IV curve. Fig 9C compares the concentration dependence of the glutamate response of coexpressed CrGluCl-C and CrGluCl-B together with the response of the individual subunits. A Hill fit to the response of the combined subunits yielded an EC_50_ of 53 ± 18.8 μM and n_H_ of 1.9 ± 0.6 (means ± SD, n = 13), an increase in sensitivity in comparison to that of CrGluCl-B but similar to that of CrGluCl-C assayed separately. Concerning ivermectin activation, CrGluCl-C and CrGluCl-B coexpressed receptor was poorly sensitive in contrast the high sensitivity of CrGluCl-C and more in line with the insensitivity of the CrGluCl-B subunit. The responses to 3 mM glutamate and 1 μM ivermectin are compared in Fig 9D. CrGluCl-C and CrGluCl-B on their own had glutamate-evoked currents that did not differ significantly from one another, whilst the current of coexpressed subunits was significantly smaller than that of CrGluCl-B (P<0.05 as tested by ANOVA). While CrGluCl-C is activated by ivermectin (P<0.05 by ANOVA compared with CrGluCl-B), there was no difference between the coexpressed C and B subunits and CrGluCl-B on its own. The small response of the coexpressed CrGluCl-C/CrGluCl-B was nevertheless significantly different from zero (P<0.001 in a one sample t-test).

Oocytes microinjected with CrGluCl-C and CrGluCl-A responded to glutamate developing currents exhibiting steady as well as transient components (Fig 9E), suggestive of a sum of the activities of the individual subunits acting separately. The experiment also shows the irreversible response to ivermectin expected for either GluCl individually expressed as well as the sensitivity to PTX. A summary of the magnitude of the responses to 3 mM glutamate and 1μM ivermectin is shown in Fig 9F. IV relations in Fig 9G show that after activation by glutamate the current at the plateau of the response is outwardly rectified as that for CrGluCl-A on its own, whilst the behaviour turns to ohmic when the peak response is plotted, reminiscent of that of CrGluCl-C expressed alone. Fig 9H shows an experiment where the dose-response of the effect of glutamate on currents at 60 and -80 mV of the coexpressed CrGluCl-A and -C was assayed. Glutamate produced a graded effect, inducing currents with increasing desensitisation as the concentration increased, an effect that was more marked at the negative potential at which the putative contribution of CrGluCl-C subunits should predominate. Fig 9I shows dose-response curves taken at 60 and -80 mV to which Hill equations were fitted. At 60 mV a single Hill equation sufficed to describe the data adequately with an EC_50_ of 7.9 ± 0.9 μM (Mean ± SD, n = 4). The data at -80 mV were better described by double Hill equations that yielded EC_50_ values of 7.5 ± 2.0 and 79 ± 16.7 μM (Mean ± SD, n = 4), approaching those of the -A and -C subunits expressed on their own (7 and 67 μM, see also Table 2). These results suggest that after microinjection of subunits A and C cRNAs the corresponding expressed receptors function independently.

### CrGluCl-D in coinjection with CrGluCl-B, CrGluCl-C or CrGluCl-A

**Fig 10:**
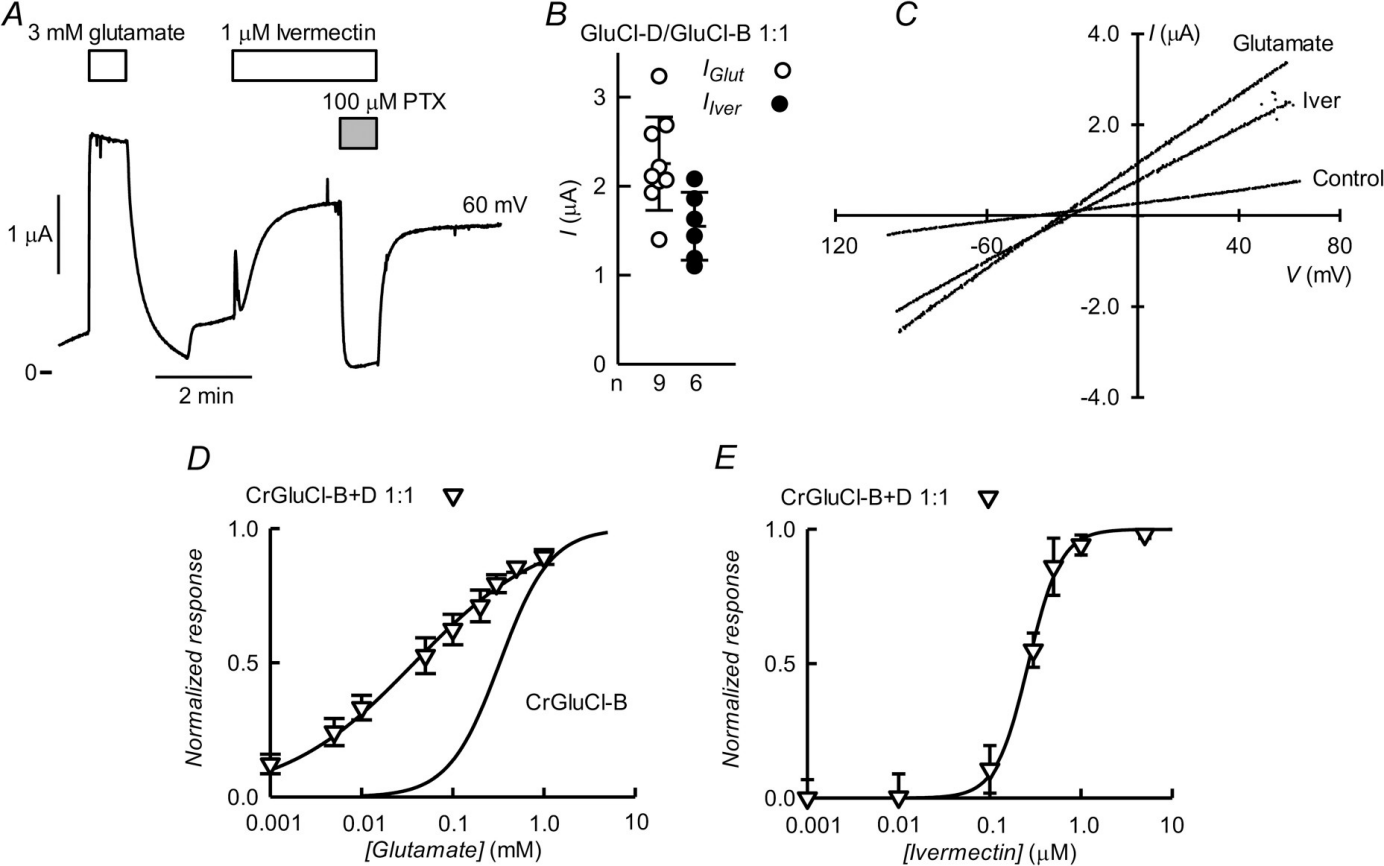
Functional properties of coexpressed CrGluCl-D and CrGluCl-B in *Xenopus* oocytes. A. Currents elicited by 3 mM glutamate or 1 μM ivermectin in an oocyte expressing subunits D and B 48–72 h after microinjection of 10ng cRNA for each CrGluCl-D and CrGluCl-B. Recording taken at 60 mV. B. Summary of currents elicited by glutamate or ivermectin in oocytes expressing CrGluCl-D and CrGluCl-B in 1:1 proportion. Total cRNA injected was always 20 ng. Individual data and means ± SDs are shown with n values given under the abscissa. C. IV relations corresponding to control before any addition or during the glutamate or ivermectin challenges. D. Dose-response relationship of the glutamate-sensitive currents of CrGluCl-D:CrGluCl-B mixed expression experiments. Data are normalized (mean ± SD, n = 6) to the maximal effect of glutamate. The line is a Hill equation fit. Data for CrGluCl-B individual expression from Fig 3 is shown for comparison. E. Dose-response relationship of the ivermectin-sensitive currents of CrGluCl-D:CrGluCl-B mixed expression experiments. Data are normalized (mean ± SD, n = 6) to the maximal effect of glutamate. The line is a Hill equation fit.

As reported above (Fig 7), expression of CrGluCl-D in oocytes gives rise to large spontaneous currents lacking sensitivity to glutamate or ivermectin but inhibited by PTX. When coinjected with CrGluCl-B in a 1:1 ratio, however, there was only a residual current consistent with the background conductance of uninjected oocytes. Oocytes coinjected with CrGluCl-D and CrGluCl-B cRNAs responded to glutamate or ivermectin addition with prompt increases in PTX-sensitive current (Fig 10A). Fig 10B shows a comparison of the currents elicited by 3 mM glutamate or 1 μM ivermectin of the D and B 1:1 mixture. IV relations for the stimulated currents were linear and reversed at potentials depolarized with respect to preaddition control IV (Fig 10C). The concentration-dependence of the glutamate effect of the coexpressed subunits (Fig 10D) has an EC_50_ of 36.5 ± 0.08 μM and n_H_ of 0.63 ± 0.11 (mean ± SD, n = 6). Concentration-dependence of the ivermectin effect on the coexpressed receptors (Fig 10E) has an EC_50_ of 278 ± 53 nM and n_H_ value of 2.6 ± 1.1 (mean ± SD, n = 6).

**Fig 11:**
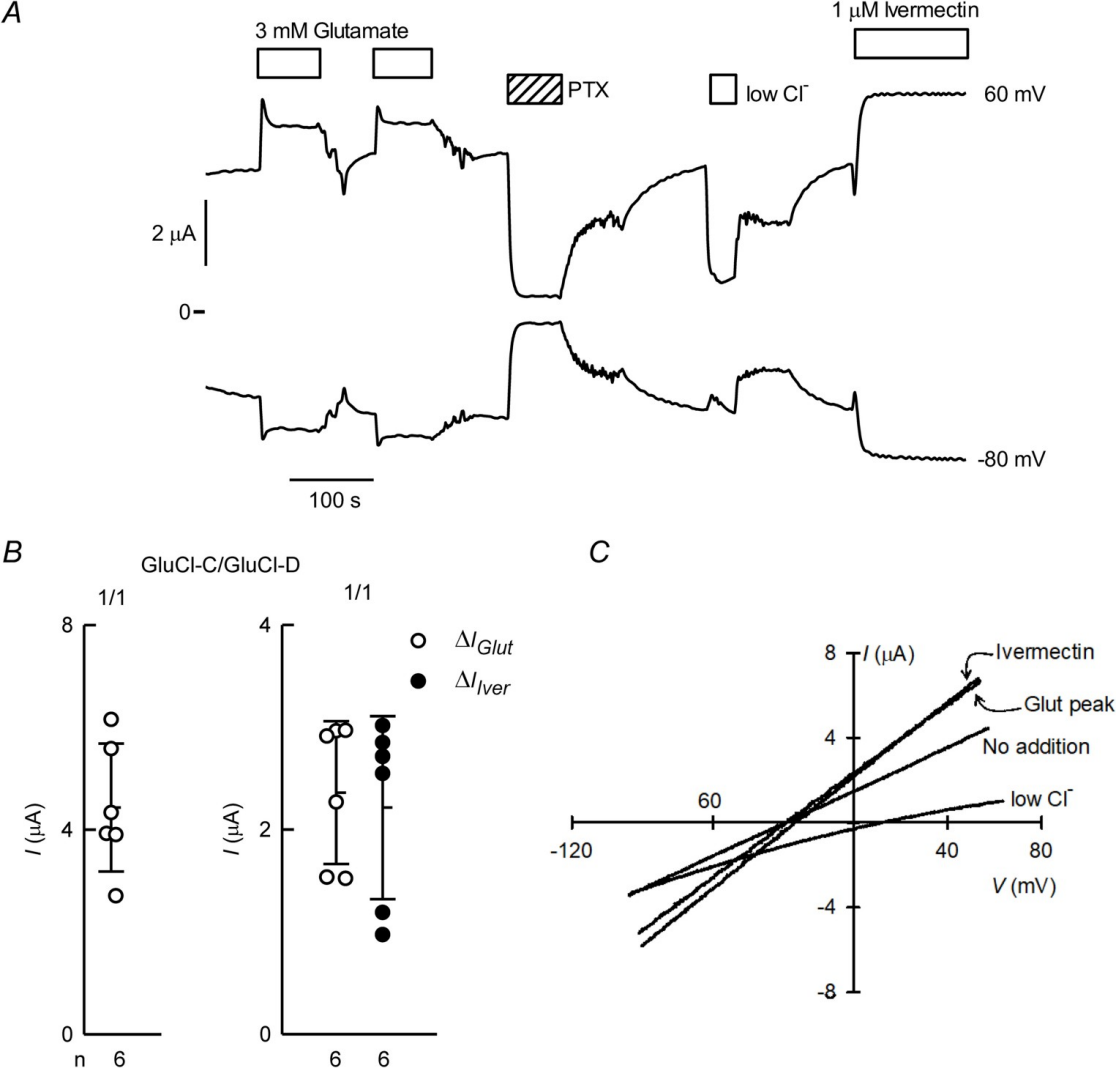
Functional properties of CrGluCl-D and CrGluCl-C expressed in *Xenopus* oocytes. A. Spontaneous currents in an oocyte expressing subunits D and C after microinjection of 10 ng cRNA for each CrGluCl-D and CrGluCl-C. The effects of 3 mM glutamate or 1 μM ivermectin, as well as that of partial chloride removal and PTX addition are also shown. Recording shown were taken at 60 mV and -80 mV. B. Summary of spontaneous currents (at 60 mV) in oocytes expressing CrGluCl-D and CrGluCl-C subunits (left) together with data for the extra current elicited by glutamate or ivermectin (right). Total cRNA injected was always 20 ng. Data points report individual measurements with means ± SDs also shown, n indicated under the abscissa. C. IV relations corresponding to the spontaneous currents before any addition (No addition), at the peak of the glutamate response, after ivermectin challenge and after partial chloride removal or PTX inhibition.

In contrast with the evidence for the formation of mixed GluCl subunits when coinjecting CrGluCl-D and CrGluCl-B cRNAs, it appears this is not the case when CrGluCl-D cRNA is microinjected together with that of CrGluCl-C. Indeed Fig 11A shows that oocytes expressing CrGluCl-D and -C exhibit the large PTX-inhibitable spontaneous currents expected for the -D subunit expressed on its own. Addition of glutamate or ivermectin elicited additional current, with that responding to glutamate exhibiting a transient component possibly mediated by CrGluCl-C subunits functioning independently. Fig 11B shows the magnitude of both the spontaneous, chloride-dependent current and the increases due to glutamate or ivermectin addition. IV curves in Fig 11C approach ohmic behaviour in the absence of stimulation and after glutamate or ivermectin challenge. The depolarization upon partial extracellular chloride removal, from -32 to 12 mV, shows the anion as the charge carrier. The mean ± SD of P_Gluconate_/P_Cl_ permeability ratio deduced from these shifts in E_rev_ was 0.15 ± 0.04 (n = 4).

**Fig 12:**
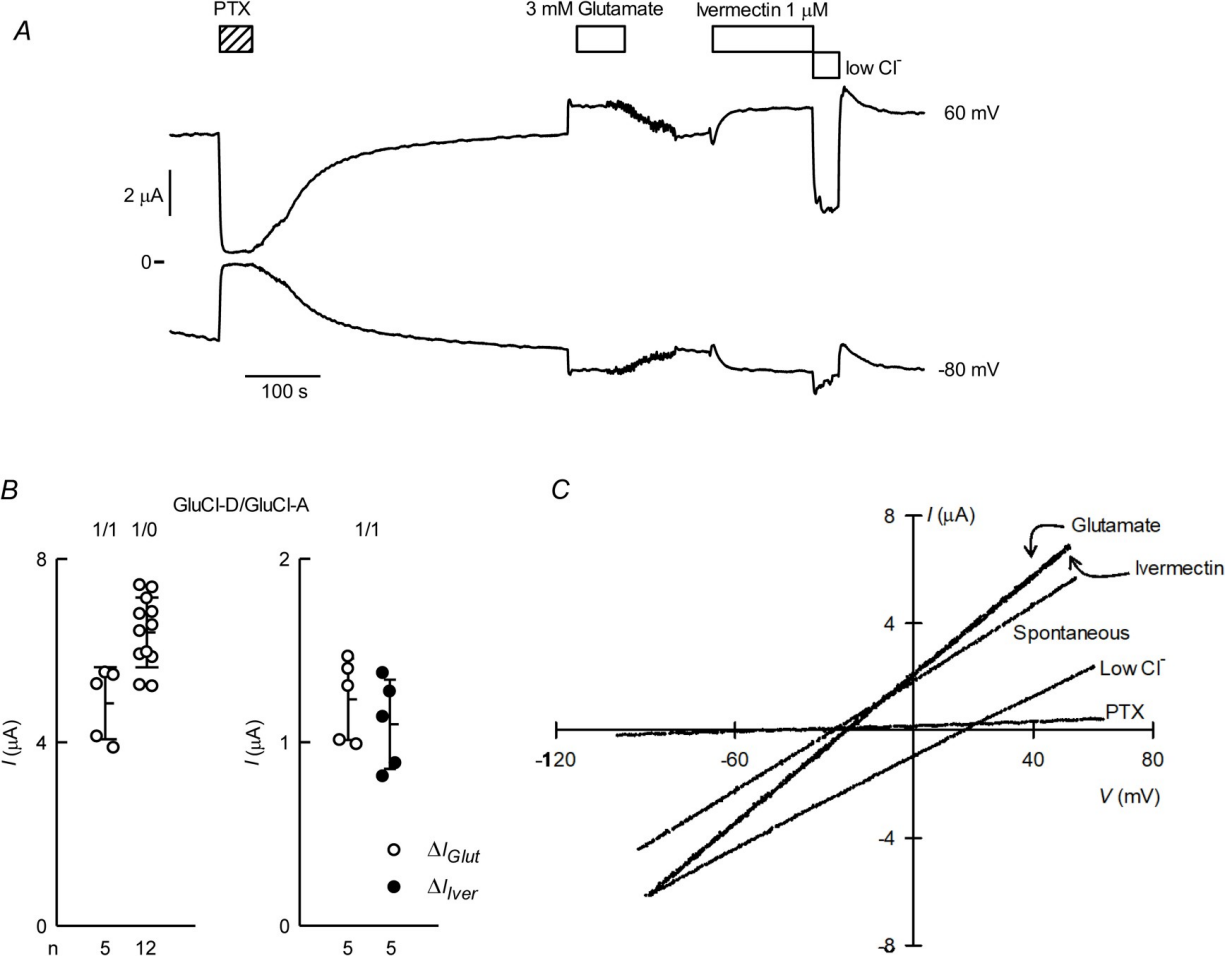
Currents in *Xenopus* oocytes microinjected with CrGluCl-D and CrGluCl-A cRNAs. A. Spontaneous currents in an oocyte expressing subunits D and A after microinjection of 10ng cRNA for each CrGluCl-D and CrGluCl-A. The effects of 3 mM glutamate or 1 μM ivermectin, as well as that of partial chloride removal are also shown. Recording shown were taken at 60 mV and -80 mV. B. Summary of spontaneous currents for the mixed D:A subunit expressions compared with those for the D subunit on its own. On the right hand panel are data for the extra current elicited by glutamate or ivermectin in oocytes expressing CrGluCl-D and CrGluCl-A. Total cRNA injected was always 20 ng. Data points report individual measurements with means ± SDs also shown, n indicated under the abscissa. C. IV relations corresponding to the spontaneous currents before any addition, during the glutamate or ivermectin challenges, after partial chloride removal or PTX inhibition.

Oocytes coinjected with CrGluCl-D and CrGluCl-A exhibit sizable chloride-dependent spontaneous currents similar to those of CrGluCl-D alone (Fig 12A). Fig 12B (left-hand panel) compares the magnitude of spontaneous currents of oocytes microinjected with cRNA for CrGluCl-D with those with D and A CrGluCl subunits. Unlike what is seen with singly expressed CrGluCl-D, glutamate or ivermectin increased the basal spontaneous current (Fig 12B, right-hand panel). IV curve for spontaneous current shown in Fig 12C approaches ohmic behaviour as do those seen after glutamate or ivermectin simulation. Depolarization in low chloride solution, from -27 to 16 mV, shows the anion as the charge carrier. The mean ± SD of P_Gluconate_/P_Cl_ permeability ratio deduced from these shifts in E_rev_ was 0.19 ± 0.03 (n = 3).

### CrGluCl-E in coinjection experiments with CrGluCl-A, CrGluCl-B or CrGluCl-C

CrGluCl-E appears not to elicit any glutamate receptor activity when expressed in *Xenopus* oocytes. We tested whether coinjection with CrGluCl-A, CrGluCl-B or CrGluCl-C might promote activity with properties different from those seen of the subunits expressed singly.

**Fig 13:**
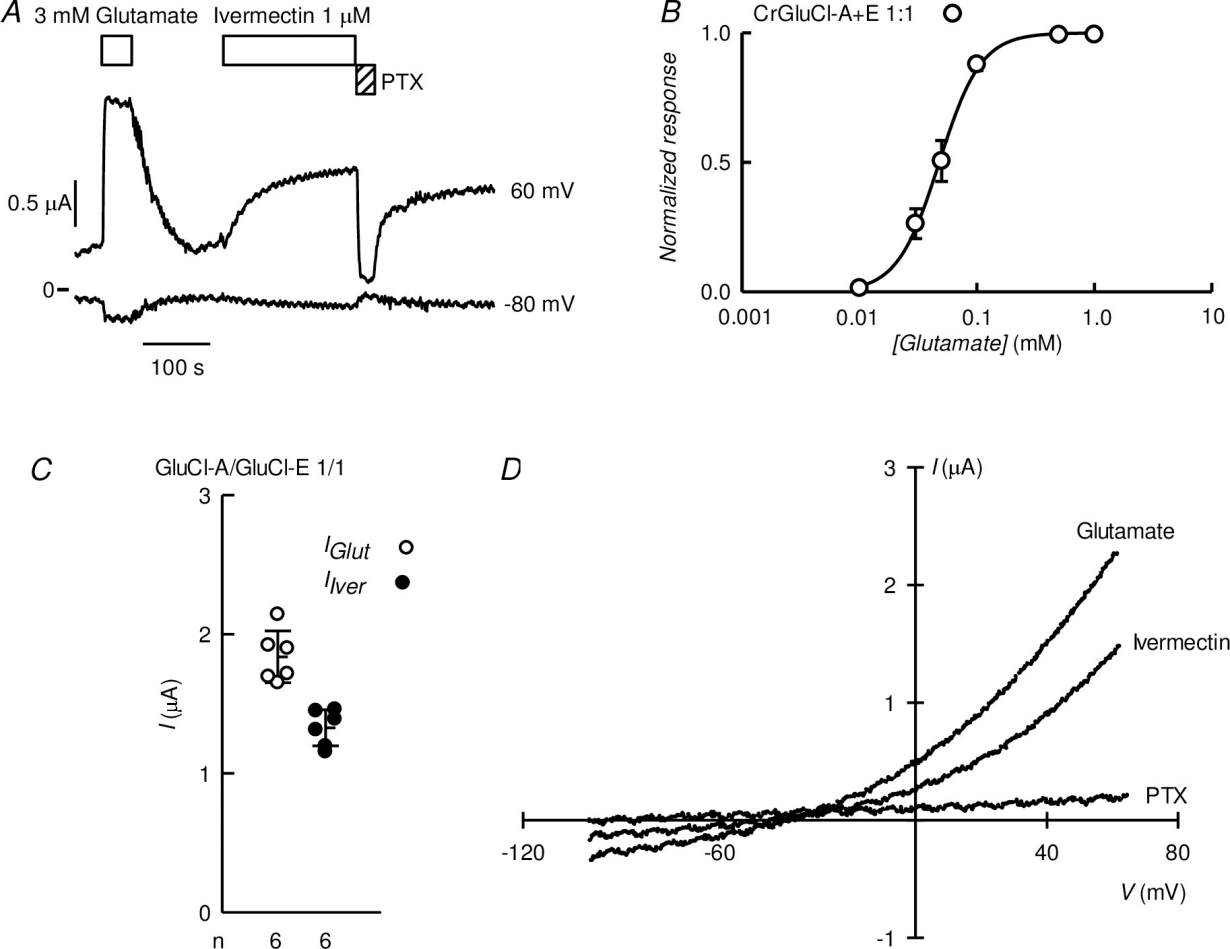
Currents in *Xenopus* oocytes microinjected with CrGluCl-E and CrGluCl-A cRNAs. A. Currents elicited by 3 mM glutamate or 1 μM ivermectin in an oocyte after microinjection of 10ng cRNA for each CrGluCl-E and CrGluCl-A. Recording taken at 60 and -80 mV. B. Dose-response relationship of the glutamate-sensitive currents of CrGluCl-EnnCrGluCl-A mixed expression experiments. Data are normalized (mean ± SD, n = 4) to the maximal effect of glutamate. The line is a Hill equation fit. C. Summary of currents elicited by glutamate or ivermectin in oocytes expressing CrGluCl-E and CrGluCl-A together in a 1:1 proportion. Total cRNA injected was always 20 ng. Data points report individual measurements with means ± SDs also shown, n indicated under the abscissa. D. IV relations corresponding to currents after glutamate or ivermectin challenges, as well as during addition of PTX.

Additional expression of CrGluCl-E does not appear to affect CrGluCl-A activity. Oocytes microintected with cRNA for CrGluCl-E and CrGluCl-A responded to glutamate and ivermectin (Fig 13A and 13C) much as expected from singly expressed CrGluCl-A. The glutamate response (Fig 13B) had an EC_50_ value of 5.0 ± 0.5 μM and n_H_ of 2.5 ± 0.3 (means ± SDs, n = 4), a sensitivity that is close to that of CrGluCl-A expressed on its own [34]. Similar to CrGluCl-A expressed alone, the IV relations of these mixed subunit experiments were markedly outwardly rectified (Fig 13D).

**Fig 14:**
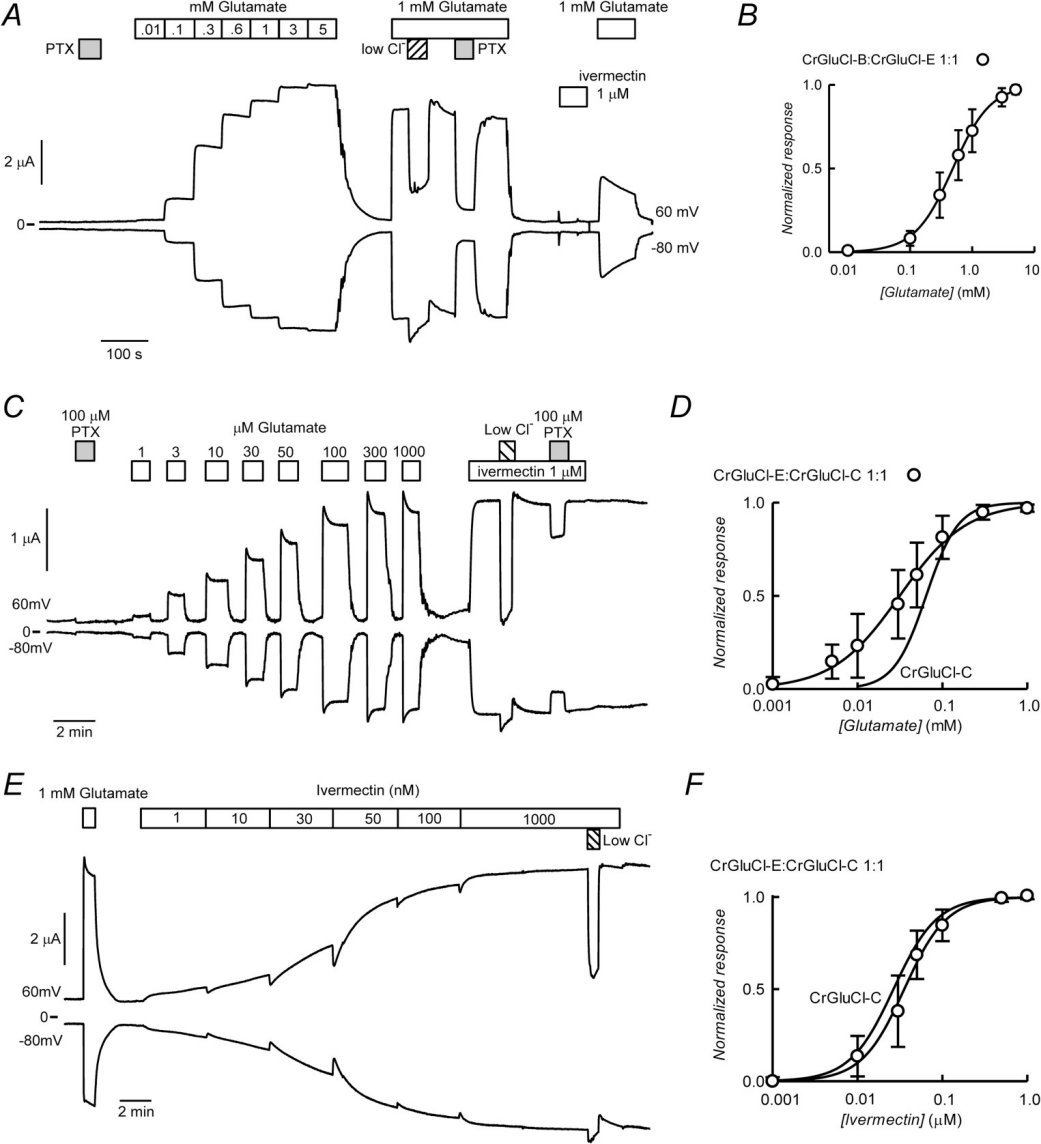
Functional properties of coexpressed subunits CrGluCl-E with CrGluCl-B and CrGluCl-E with CrGluCl-C in *Xenopus* oocytes. A. Currents elicited by increasing glutamate concentrations in an oocyte expressing subunits E and B after microinjection of 10 ng cRNA for each CrGluCl-E and CrGluCl-B. Recording taken at 60 and -80 mV. Also shown are the effect of partial chloride removal and addition of 100 μM PTX. Ivermectin addition was done at 1μM. B. Dose-response relationship of the glutamate-sensitive currents of CrGluCl-E:CrGluCl-B mixed expression experiments. Data are normalized (mean ± SD, n = 6) to the maximal effect of glutamate. The line is a Hill equation fit. C. Currents elicited by increasing glutamate concentrations as indicated or 1 μM ivermectin in an oocyte expressing subunits E and C 48–72 h after microinjection of 10ng cRNA for each CrGluCl-E and CrGluCl-C. Recordings taken at 60 and -80 mV. D. Dose-response relationship of the glutamate-sensitive currents of CrGluCl-E:CrGluCl-C mixed expression experiments. Data are normalized (mean ± SD, n = 6) to the maximal effect of glutamate. The line is a Hill equation fit. That for CrGluCl-C expressed on its own is also shown for comparison. E. Currents elicited by increasing ivermectin concentrations as indicated in an oocyte microinjected with 10ng cRNA for each CrGluCl-E and CrGluCl-C. F. Dose-response relationship of the ivermectin-sensitive currents of CrGluCl-E:CrGluCl-C 1:1 mixed expression experiments. Data are normalized (mean ± SD, n = 5) to the maximal response to ivermectin. The line is a Hill equation fit. That for CrGluCl-C expressed on its own is also shown for comparison.

Additional expression of CrGluCl-E did not affect CrGluCl-B activity markedly. Superfusion with increasing concentrations of glutamate of an oocyte expressing CrGluCl-E and CrGluCl-B in a 1:1 ratio led to a graded increase in current with no evidence for desensitization (Fig 14A). Reduction in extracellular chloride caused a marked decrease in outward current while addition of channel blocker PTX at 100 μM strongly inhibited both inward and outward currents. In contrast with the glutamate response, ivermectin was without any activating effect, nevertheless a subsequent challenge with 1 mM glutamate was markedly antagonised. Fig 14B shows the dose dependence of the glutamate effect on the 1:1 mixed subunits measured at 60 mV. The relationship can be described by a Hill equation and the average response had an EC_50_ value of 534 ± 239 μM and n_H_ of 1.51 ± 0.08 (means ± SDs, n = 6), which compares with respective values of 388 ± 203 μM and 1.7 ± 0.1 (means ± SD, n = 16) for subunit B on its own. The EC_50_ values did not differ (P = 0.08 by t-test), but the difference between n_H_ values did reach statistical significance (P<0.05)

Assaying glutamate response after coinjection of cRNA for CrGluCl-E with that of the CrGluCl-C subunit gave rise to dose-dependent robust currents that unlike those of CrGluCl-C on its own showed little desensitization (Fig 14C). The concentration dependence of the response to glutamate (Fig 14D) followed a Hill equation with EC_50_ of 36.5 ± 22.5 μM and n_H_ of 1.4 ± 0.4 (means ± SDs, n = 10). Fig 14C also shows that after glutamate removal, addition of 1 μM ivermectin elicited an irreversible increase in currents that, very much as is the case with CrGluCl-C, could only be partially inhibited by 100 μM PTX. That chloride is the charge carrier is witnessed by the decrease in outward current after extracellular Cl^-^ removal from the extracellular solution.

Ivermectin activation of the anion current in oocytes microinjected with CrGluCl-E and CrGluCl-C cRNA is graded and carried by chloride (Fig 14E). The average response to ivermectin (Fig 14F) yielded EC_50_ and n_H_ values of 37.4 ± 13.8 nM and 2.1 ± 0.67 (means ± SD, n = 5), very close to those for CrGluCl-C assayed on its own (31.1 nM and 2.1 respectively).

### Structural basis for the lack of ivermectin activation of CrGluCl-B

**Fig 15:**
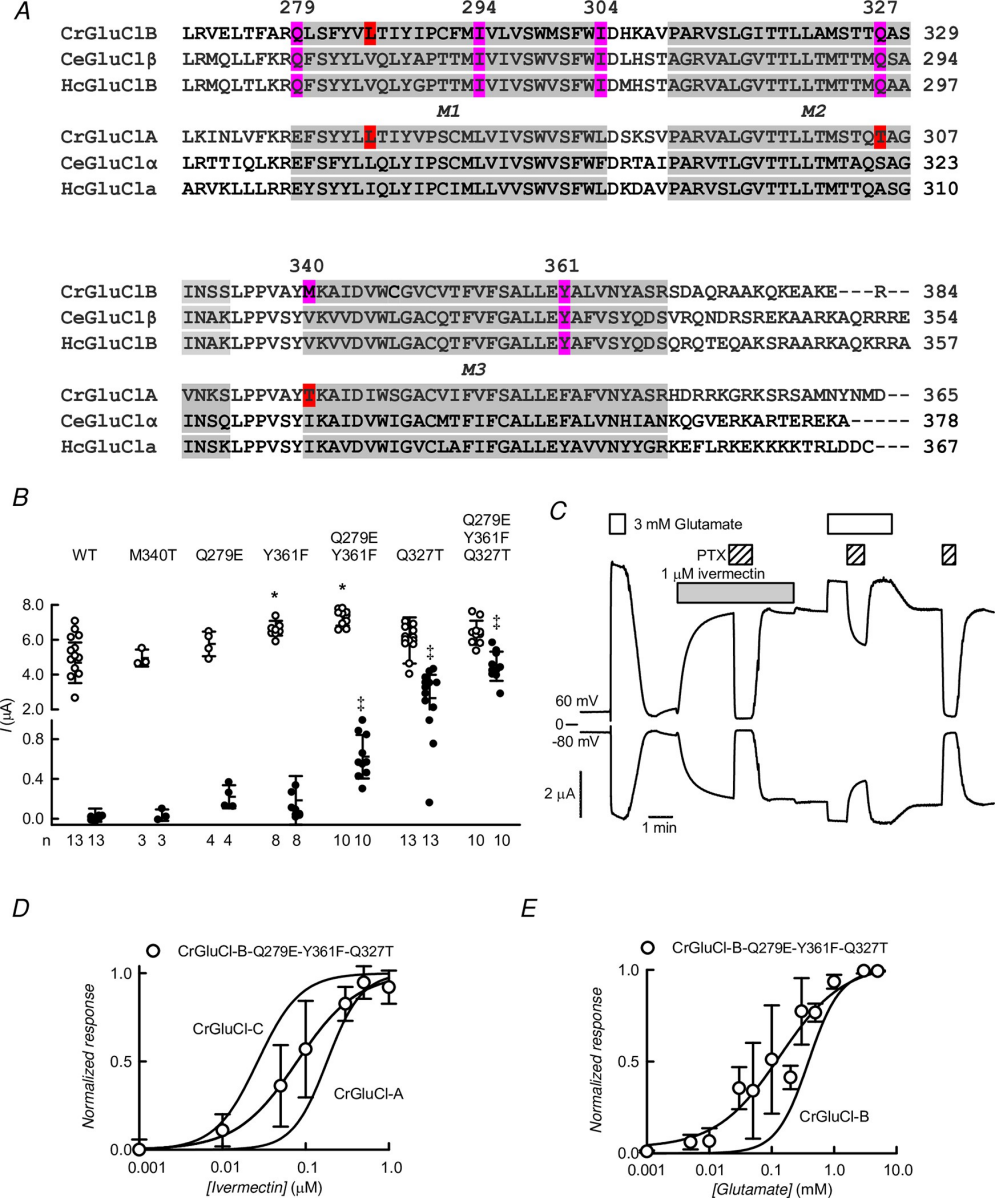
Mutational analysis of CrGluCl-B ivermectin-resistance. A. Partial sequence alignment of CrGluCl-B and CrGluCl-A. Also shown are homologous regions of GluCl α and β subunits of *H*. *contortus* and *C*. *elegans*. Highlighted in red are residues identified as interacting with ivermectin in docking experiments with CrGluCl-A. Magenta residues are those at variance between *C*. *rogercresseyi* GluCl A and B subunits and are conserved in the nematode respective subunits. B. Currents induced in CrGluCl-B WT or its indicated mutants by 3 mM glutamate (open circles) or 1 μM ivermectin (black circles). Results are given as individual points and means ± SD given under the abscissa for WT CrGluCl-B and for its M340T, Q279E, Y361F, Q279E-Y361F, Q327T and Q279E-Y361F-Q327T mutants. ANOVA with Bonferroni correction analysis of the glutamate effect shows no significant difference between mutants and WT except as indicated by * (P<0.001). Regarding ivermectin effect, one-sample t-tests showed significant effects of the drug in all cases, except for the WT receptor and for its M340T mutant. The effect of ivermectin on the mutants compared with that on the WT subunit reached significance as tested by ANOVA as indicated (‡, P<0.05). C. Currents taken at 60 and -80 mV in a recording of the Q279E-Y361F-Q327T mutant. D and E. Concentration-dependence of the effects of ivermectin and glutamate on CrGluCl-B-Q279E-Y301F-Q327T triple mutant. Concentration-dependence data for ivermectin (panel D) are means ± SD with n = 8 and were normalised to the maximal effect evoked by ivermectin after a Hill equation was fitted to the individual data sets. Fitted parameters were EC_50_ 94 ± 54 nM and n_H_ 1.6 ± 0.22 (means ± SD). Curves describing ivermectin effect on CrGluCl-A and -C are also shown. E. Normalised response to glutamate of the triple mutant after fitting a Hill equation to the individual data sets is given as mean ± SD (n = 21). Average parameters were EC_50_ 103 ± 133 μM and n_H_ 1.6 ± 0.22 (means ± SD). The fitted curve for CrGluCl-B dose response is also shown.

Analysis of sequence differences between CrGluCl-B, refractory to ivermectin activation, and the ivermectin-sensitive subunit CrGluCl-A was focused on transmembrane segments M1-M3 that harbour points of contact of the drug in the CeGluClα structure [15]. Fig 15A shows sequence alignments of CrGluCl-A and -B with respectively ivermectin-sensitive and -insensitive α and β GluCl subunits of *C*. *elegans* and *H*. *contortus*. Six residues, highlighted in magenta in Fig 15A, differ between the *C*. *rogercresseyi* subunits. Three amino acids located in transmembrane domains M1, 2 and 3 respectively have been implicated in ivermectin binding to CrGluCl-A: L263, T305 and T315 (identified in red in Fig 15A [34]). L263 is conserved in CrGluCl-B and two other residues, I294 and I304, experience conservative changes to L in subunit A, and were therefore not considered further. In contrast, T305 and T315 are the non-conserved Q327 and M340 in the CrGluCl-B subunit. Further non-conservative differences in CrGluCl-B are Q279 and Y361. These residues were mutagenized in CrGluCl-B to those present in subunit -A to ascertain their role in ivermectin sensitivity.

The response to glutamate of the mutants (Fig 15B) was not significantly different from that of the WT receptor, except for Y361F and double Q279E-Y361F mutants, (ANOVA P<0.001). Ivermectin, on the other hand, had no effect on WT or M340T mutant, but produced small but significant effects on Q279E, Y361F and a double mutant carrying both modifications (one-sample t-tests). The effect of ivermectin on the singly Q327T mutated subunit, as well as that on the double and triple mutant were all significantly greater than those of the WT GluCl (ANOVA P<0.05). Currents elicited by 3 mM glutamate or 1 μM ivermectin in an oocyte expressing CrGluCl-B-Q279E-Y361F-Q327T triple mutant taken at 60 and -80 mV are shown in Fig 15C. The response to ivermectin of this triple mutant is irreversible and the activated current is sensitive to receptor blocker PTX. Dose response experiments (Fig 15D) confirm the ivermectin sensitivity of the triple mutated CrGluCl-B. Hill equations fitted to the individual data sets gave parameters of EC_50_ 94 ± 54 nM and n_H_ 1.6 ± 0.22 (means ± SD), intermediate between those of the -C and -A subunits. Glutamate response occurs with significantly higher sensitivity (Fig 15E). Hill equation analysis of the data yielded average parameters of EC_50_ 103 ± 133 μM and n_H_ 1.6 ± 0.22 (means ± SD).

## Discussion

Avermectins such as ivermectin have been highly successful antiparasitic agents used widely in human and veterinary medicine. Ivermectin has been of central importance in the strategy to eliminate onchocerciasis, lymphatic filariasis, and strongyloidiasis (reviewed in [41]). Ivermectin and other avermectins are also important in the context of parasitic diseases affecting livestock and agricultural productivity [9]. In fish aquaculture, sea lice infestation was initially successfully controlled using avermectins, but more recently resistance to the drugs has developed by mechanisms that remain unclear [42]. The target of avermectins are GluCl receptors that are irreversibly activated by the drug thus conducing to parasite paralysis and death. The possibility that mutation of these ligand-gated chloride channels is responsible for resistance has been considered. Alternatively, the expression of naturally existing avermectin activation-resistant receptor subunits could also be responsible for parasite evasion of the pesticidal agents. Exploration of these possibilities requires a so far lacking detailed knowledge of the GluCls present in sea lice. In this report we identify and functionally characterise four novel GluCls subunits in *Caligus rogercresseyi* that together with a previously reported subunit [34] constitute a family of five receptor subunits encoded by different genes. Functional assays in *Xenopus* oocytes show that of the five glutamate-gated chloride receptors discussed three, CrGluCl-A, CrGluCl-B and CrGluCl-C, present bona fide receptor behaviour when expressed on their own. Expression of CrGluCl-D leads to constitutive chloride current consistent with spontaneously active GluCl channels, whilst CrGluCl-E did not present any activity upon stimulation with glutamate or ivermectin. Details of these results are summarised in Table 2 to facilitate comparisons between subunits.

**Table 2:** Summary of glutamate and Ivermectin effects on CrGluCl subunits.

Subunit	EC_50_ Glutamate (μM)	n_H_	EC_50_ Ivermectin (nM)	n_H_	
GluCl-A	7.2 ± 2.7	1.65 ± 0.63	181 (*)	2.1	
GluCl-B	388 ± 203	1.7 ± 0.1	Insensitive	-	
GluCl-C^*a*^	67 ± 25	2.5 ± 0.56	31 ± 14	2.1 ± 0.67	
GluCl-D^*b*^	NM	-	NM	-	
GluCl-E^*c*^	NM	-	NM	-	
**Coexpressed subunits**	**Ratio**	**EC**_**50**_ **Glutamate (μM)**	**n**_**H**_	**Iver response to activation (nM)**	**n**_**H**_
**A:B**	1:1	82 ± 35	0.88 ± 0.19	Insensitive	
**A:B**	4:1	89 ± 47	0.68 ± 0.10	Insensitive	
**A:B**	1:4	72 ± 47	1.08 ± 0.16	Insensitive	
**B:C** ^*d*^	1:1	53 ± 18.8	1.9 ± 0.6	Insensitive	
**B:D** ^*e*^	1:1	38.3 ± 25.7	0.62 ± 0.12	278 ± 53	2.6 ± 1.1
**B:E** ^*f*^	1:1	534 ± 239	1.51 ± 0.08	Insensitive	
**C:E** ^*g*^	1:1	36.5 ± 22.5	1.4 ± 0.4	37.3 ± 13.7	2.1 ± 0.7
**C:A** (60mV)	1:1	7.9 ± 0.9	1.2 ± 0.3	Sensitive	
**C:A** (-80mV)	1:1	7.6 ± 2.0	2.2 ± 0.4	Sensitive	
		79 ± 16.7	2.1 ± 0.8		
**D:C**	1:1	NM	-	Sensitive	
**D:A**	1:1	NM	-	Sensitive	
**E:A** ^*h*^	1:1	5.0 ± 0.5	2.5 ± 0.3	Sensitive	

NM: not measurable

*a*: desensitises during glutamate activation

*b*: spontaneously open

*c*: no activity detected

*d*: loses glutamate desensitisation

*e*: not spontaneously open

*f*: not different from B subunit alone

*g*: glutamate desensitisation absent

*h*: not different from A subunit alone

(*): from ref 34

CrGluCl-A and CrGluCl-C show highest sensitivity to glutamate, with respective EC_50_ values of ~7 and ~70 μM. Receptors with similar sensitivities to glutamate have been reported in other species of arthropods or helminths. The high sensitivity of CrGluCl-A to glutamate though unusual is similar to that obtained for *Schistosoma mansoni* SmGluCl3 [43] and *Haemonchus contortus* HcGluCla [44]. Examples of GluCls with glutamate EC_50_ values in the tens of μM, as that of CrGluCl-C, are *C*. *elegans* CeGluClα [36], *Drosophila melanogaster* DmGluClα [39], *Haemonchus contortus* HcGluClα [37,45], *Musca domestica* MdGluClα [46] and *Cooperia oncophora* CoGluClα3S [47]. CrGluCl-B, with an EC_50_ of ~400 μM, has the lowest glutamate affinity of the *C*. *rogercresseyi* subunits. Examples of other GluCls with high glutamate EC_50_ values in the hundred μM, or even in the mM, range are *C*. *elegans* CeGluClα2A [48] and CeGluClα4 (GLC-3) [49], and *Haemonchus contortus* HcGluClβ [37].

CrGluCl-A and CrGluCl-C are activated by ivermectin, but CrGluCl-B is completely insensitive to activation by this antiparasitic macrocyclic lactone. Despite the failure to activate CrGluCl-B, both ivermectin and emamectin antagonise the effect of glutamate such that the response to the agonist is decreased after treatment with the drug. This phenomenon was observed using CeGluClβ (also called CeGLC-2) subunits [50]. The inhibition of glutamate activated current in CeGLC-2 occurs with IC_50_ and n_H_ values 1.28 μM and -1.74 [51], which compares with figures of 0.34 μM and -2.2 measured here for CrGluCl-B. Notice that we assume complete reversibility of PTX action. This is verified in the case of the subsequent glutamate response (see e.g. Fig 4A and 4B), but it has not been formally demonstrated for the avermectin effect.

Concerning activation of GluCls by avermectins, whilst CeGluClβ does not activate in response to ivermectin and other macrocylclic lactones, CeGluClα is activated by the drug [36,51]. HcGluClα and HcGluClβ are respectively sensitive and insensitive to ivermectin [37]. Coexpression of the α and β subunits of both *C*. *elegans* and *H*. *contortus* GluCls leads to heteromeric channels activated by both glutamate and ivermectin, but while the inclusion of the β subunit does not change the potency of ivermectin in *C*. *elegans* coexpression, the potency and efficacy of ivermectin in HcGluCl-α/β coexpression are diminished [37]. Remarkably however, coexpression of CrGluCl-B with either CrGluCl-A or CrGluCl-C gives rise to channels that, in addition to being more sensitive to glutamate than CrGluCl-B expressed singly, are not activated by ivermectin (Table 2). These results show that these subunits can form heteromeric assemblies with emerging new properties such as intermediate glutamate sensitivities but, importantly, they show that CrGluCl-B can confer these heteromers its refractoriness to activation by ivermectin.

The failure of the ivermectin activating effect is also present in experiments using a 1:1 expression ratio of CrGluCl-B and CrGluCl-A. If the assembly of these heteromers occurred randomly, a binomial expansion-based calculation would suggest only a 3% of homopentameric receptors implying that a single CrGluCl-B subunit would be necessary to render the pentamers non-activated by ivermectin. Surprisingly, a similar result is obtained when oocytes are injected with the subunit ratios of 1:4 and 4:1. This would imply a preferential assembly as complexes containing at least one CrGluCl-B subunit. This would be in line with results obtained using heteromeric *C*. *elegans* GluClα and β subunits which are shown to assemble with a fixed stoichiometry [14].

Two other subunits described here do not elicit regular CrGluCl receptor behaviour upon functional assay. CrGluCl-D generates what might be considered spontaneously active GluCl behaviour while no currents are observed when expressing CrGluCl-E, perhaps suggesting it does not reach the membrane surface. That they can both function as subunits of distinct heteromeric assemblies is suggested by coinjection experiments. Indeed, coexpressing CrGluCl-D together with CrGluCl-B leads to channels that are closed in the absence of either glutamate or ivermectin but that readily activate in their presence. Interestingly receptors arising from CrGluCl-D and CrGluCl-B coexpression, in contrast to subunit B on its own or in combination with other subunits, are sensitive to ivermectin and have high sensitivity to glutamate (Table 1). We have no explanation for this. Examination of predicted ivermectin binding sites of the subunits reveals high conservation along all five *Caligus* receptors. The only exception is present in CrGluCl-D M3 where Gly323 replaces a threonine in subunits B, C and E, and isoleucine in A, but we have not tested the functional consequence of this replacement.

CrGluCl-E shows no channel activity on its own and appears not to affect the behaviour of CrGluCl-B in coinjection experiments. Expressing together subunits E and C gives rise to channels responding to glutamate and ivermectin similarly to CrGluCl-C on its own. These heteromeric channels, however, differ in that the characteristic CrGluCl-C desensitising response to glutamate is absent when CrGluCl-E is expressed together with CrGluCl-C, in which case the resulting channel possesses sustained currents in the presence of the agonist. In contrast to the association of CrGluCl-A and -B, CrGluCl-A does not appear to give rise to new heteromeric assemblies with subunits C, D or E. We cannot discard, however, that heteromers between these subunits might form with very similar rectification properties, EC_50_ and n_H_ values to those of CrGluCl-A on its own. Future studies with ivermectin and other agonists might help to elucidate this point further.

Interestingly, a recent paper [38] describes a variety of GluCl subunits in the parasitic nematodes *Brugia malayi* and *Parascaris univalens* which are orthologues to *C*. *elegans* receptors AVR-14B, GLC-2, GLC-3 and GLC-4. Functional expression of these reveals no or very low activity as GluCls when expressed on their own. Robust glutamate-activated responses can be seen, however, in coexpression experiments. These data, together with our observations of conventional receptor behaviour of heteromers including subunits CrGluCl-D or CrGluCl-E, suggest that subunit integration into mixed pentameric assemblies might be of high relevance to the expression of otherwise inactive subunits as functional glutamate receptors.

An important challenge for future research on the GluCl receptors of *C*. *rogercresseyi* (and *L*. *salmonis*) is that of whether subunit assemblies formed *in vitro* by the recombinant proteins represent receptors that are present in the copepod. This uncertainty stems from near complete lack of knowledge of many aspects of the GluCl biology, including in which cells the GluCls are expressed, at what life stage, possible sexual dimorfism in expression of the channels, among others. Another potentially important point to consider is whether, as shown for the acetylcholine receptors of *C*. *elegans* [52], there are auxiliary subunits for the GluCls that might affect their heteromerisation, localisation or activity. Work based upon imunolocalisation has been exploited to demonstrate the coexpression of certain GluCl subunits both in C. elegans and the parasitic nematodes *C*. *oncophora* and *H contortus*, but these interesting new data fall short of demonstrating actual formation of heteromeric receptors (reviewed in [53]). Future research in these aspects of the CrGluCl family should help in our knowledge of the expression patterns of the subunits in the sea louse and might help in our understanding of the mechanisms of avermectin resistance.

Exploration of the possible molecular determinants of the absence of activation of CrGluCl-B by ivermectin identified amino acids located in transmembrane domains M1-M3 that diverged from ivermectin-activated subunits such as CrGluCl-A. Mutating CrGluCl-B residues Q279, Y361 and Q327 to their homologues present in CrGluCl-A conferred ivermectin-activation to this normally refractory subunit. By far the largest contribution to this changed ivermectin sensitivity resided in Q327 of the -B subunit, which is T in the CrGluCl-A subunit. This threonine residue at position 15’ of M2 [54], has been identified in ivermectin docking simulations in CrGluCl-A as making H-bond contact with the cyclohexene ring of the drug [34]. Interestingly, recently Kaji at al. [51] have identified the homologous to Q15’ of *C*. *elegans* GluClβ (S15’ in the α subunit) as essential for the resistance to activation of this receptor to ivermectin. This residue is also conserved in ivermectin non-activable by *H*. *contortus* GluClβ [37], suggesting that perhaps a common mechanism underlies lack of receptor activation by ivermectin across species.

In the present paper we have explored the ivermectin effect on the CrGluCls, taking it as a “representative” molecule of the macrocyclic lactone family. We have shown that emamectin acts in a similar way to ivermectin on CrGluCl-A, where it appears to share identical binding site, and on CrGluCl-B. It will be interesting to address the issue of the sensitivity the CrGluCls to an extended number of macrocyclic lactones that are known to have a diversity of effects on this type of subunit.

In summary, we have discovered a family of five GluCl subunits in the salmon fish parasite copepod *Caligus rogercresseyi*. Of the five subunits identified, and dubbed CrGluCl-A to -E, CrGluCl-B is refractory to activation by ivermectin, an important antiparasitic macrocyclic lactone. CrGluCl-B is also able to confer this refractoriness to functionally distinct heteromeric glutamate assemblies generated by its coexpression with CrGluCl-A or CrGluCl-C. This might contribute to the resistance of this parasite to conventional treatments based on macrocyclic lactones. We suggest that these *Caligus rogercresseyi* GluCl subunits, which appear highly conserved in *Lepeophtheirus salmonis*, another parasite of importance in fish aquaculture, could serve as molecular markers to assess susceptibility to existing treatments and could become useful molecular targets in the search for novel antiparasitic drugs.

## Methods

### Ethics statement

All animal procedures were approved by the Centro de Estudios Científicos (CECs) Institutional Animal Care and Use Committee and were performed following “Guidance on the housing and care of the African clawed frog, *Xenopus laevis*” recommendations from Research Animals Department–The Royal Society for the Prevention of Cruelty to Animals (http://www.rspca.org.uk/xenopus).

### Identification and cloning of glutamate activated chloride channels

To identify putative GluCl subunits, we queried the NCBI Transcriptome Shotgun Assembly Sequence (TSA) Database for “glutamate-gated chloride channel Caligus”. The obtained sequences were translated and BLASTed (tBLASTn) against the C. *rogercresseyi* genome (WGS, BioProject ID 280098, NCBI) and against transcripts of *L*. *salmonis* database (https://licebase.org/). Primers for each of the putative new subunit found were designed to span the full length of the open reading frame (ORF) for each of them (B-E) (S1 Table, and arrows in Fig 1). Complete cDNAs were generated by RT-PCR from adult *C*. *rogercresseyi* samples from which putative full-length peptide sequences of CrGluCl-B, CrGluCl-C, CrGluCl-D and CrGluCl-E were obtained. Each PCR product was cloned in pGEM-T easy vector and sequenced. To express the clones in *Xenopus laevis* oocytes, ORF of CrGluCl-B, -C, -D and -E were cloned in pGH19 vector from pGEM-Teasy/CrGluCl using the following restriction enzymes: BamHI and XbaI (-B) and EcoRI (-D and -E). CrGluCl-C was cloned first in pCR3.1 vector with NotI and then into pGH19 with SpeI/XbaI. The CrGluCl/pGH19 plasmid were linearized with NheI (-A and -B) or XhoI (-C, -D, -E) and used as template for the synthesis of capped cRNA (complementary RNA) using the mMessage Machine T7 kit [34]. Mutations were made by site-directed mutagenesis using the QuickChange method and confirmed by sequencing.

### Phylogenetic analysis

The evolutionary relationship between CrGluCl subunits and GluCls from *L*. *salmonis*, other arthropods and chosen nematodes, was studied. Phylogenetic and molecular evolutionary analyses were conducted using MEGA version 11 [55]. The sequences were aligned using the MUSCLE algorithm and the phylogenetic tree was reconstructed using the maximum likelihood method with default parameters and 100 bootstrap replicates. Reliability for internal branch was assessed using the bootstrapping method. Graphical representation and edition of the phylogenetic tree were performed using FigTree v1.4.4 software (https://github.com/rambaut/figtree/releases/tag /v1.4.4). The sequences used in this study are available on GenBank under the following accession numbers: *Caenorhabditis elegans* (Cel): AVR-14A:AAC25481.1, AVR-14B:AAC25482.1, AVR-5:CAA04170.1, GLC-1:NP_507090.1, GLC-2:NP_491470.1, GLC-3:CAB51708.1, GLC-4:NP_495489.2. *Haemonchus contortus* (Hco): GBR-2B:CAA74623.1, GLC-2:CAA70929.1, GLC-5:AAG43233.1. *Cooperia oncophora* (Con): GluClalpha3: AAR21855.1, GluClbeta: AAR21856.1. *Brugia malayi* (Bma): AVR-14B subunit: QRW38687.1, GLC-2:QRW38684.1, GLC-3:QRW38685.1, GLC-4: QRW38686.1. *Parascaris univalens* (Pun): AVR-14B:QRW38241.1, GLC-2:QRW38238.1, GLC-3:QRW38239.1, GLC-4:QRW38240.1. *Schistosoma mansoni* (Sma): GLC-1:AGV21039.1, GLC-2.1:AGV21040.1, GLC-2.2:AGV21041.1, GLC-3:AGV21042.1. *Drosophila melanogaster* (Dme): GLC-A: AAC47266.1. *Musca domestica* (Mdo): GLC-A: BAD16657.1. *Anopheles gambiae* (Aga): GLC-B1: AGS43090.1, GLC-C: AGS43092.1. *Tetranychus urticae* (Tur): GLC-3:BAJ41378.1.

### *Xenopus oocyte* preparation and cRNA injection

Adult female *Xenopus laevis* frogs were anaesthetized by immersion in a1g/L solution tricaine (Ethyl 3-aminobenzoate methanesulfonate, Sigma) buffered to pH 7 with NaHCO_3_. Surgery was performed 30–40 min later, once the frog was completely anaesthetized judging by loss of reflexes. Ovarian lobes were removed through 1 cm abdominal incision and then kept at 16°C in Barth’s modified solution containing (in mM): 88 NaCl, 1 KCl, 0.4 CaCl_2_, 0.3 Ca (NO_3_)_2_, 0.8 MgSO_4_, 2.4 NaHCO_3_, 5 Hepes, 2.5 sodium pyruvate, 100 μg/ml gentamicin sulfate, 100 U/ml penicillin, 100 μg/ml streptomycin, 250 μg/ml amphotericin B, 0.22μm filtered and adjusted to pH 7.5 with Tris-Base. Osmolarity was brought to 220 mOsm with saccharose. The oocytes were manually defolliculated on the same day of extraction and the following day 20 ng cRNA in nuclease-free water was injected into stage V-VI oocytes using a FemtoJet microinjector (Eppendorf). Finally, the injected oocytes were stored in 96 well plates containing Barth’s modified solution and kept at 16°C until experiment day.

### Voltage-clamp assay of receptor activity in *Xenopus laevis* oocytes

Current measurements at controlled membrane potentials (voltage-clamp experiments) were performed using two intracellular microelectrodes in defolliculated *Xenopus* oocytes previously microinjected with 20 ng of cRNA. Voltage-clamp recording was performed at room temperature using a TURBO TEC-10CX amplifier (npi electronic GmbH, Tamm, Germany) and PClamp 10.6 software (Axon Instruments, USA), 1 to 4 days after oocyte injection. The oocytes were recorded in a chamber (Model RC-1Z, Warner Instrumenst, USA) under continuous superfusion. The electrodes had a resistance of 0.5–2 MΩ when filled with 3 M KCl. Reference was an Ag-AgCl electrode connected to the bath via a 3 M KCl in 3% agar bridge. Currents were recorded continuously during the experiments without interruptions for solution changes. Oocytes were held at -30 mV and a voltage protocol consisting of a voltage drop to -100 mV for 25ms followed by a 360 ms ramp from -100 mV to 60mV applied with a period of 565 ms was continuously given. The signal filtered at 1 kHz was acquired with a Digidata 1440A Analog-to-Digital Converter and analyzed with Axon pClamp 10.6 software. The solution that bathed the oocytes during the electrophysiological recordings had the following composition (mM): 115 NaCl, 2 KCl, 1.8 CaCl_2_, 1 MgCl_2_, 10 HEPES pH 7.5 obtained with NaOH. For the low chloride solution, all the NaCl was replaced with the gluconate salt of Na. This decreases chloride concentration from 122.6 to 7.6 mM while increasing that of gluconate from 0 to 115 mM. The stock solutions of the drugs in dimethyl sulfoxide (DMSO) were: 10 mM emamectin, 10 mM ivermectin, 100 mM picrotoxin. These were diluted in bath solution to obtain final concentrations. The highest DMSO concentration used was found to have no effect on CrGluCl currents.

### Data analysis

To obtain the Dose-Response curves, the currents measured at the 60mV potential for each added agonist concentration (glutamate or avermectin), were extracted and a 4-parameter Hill equation (Formula 1) fit using Sigmaplot 12.3 was performed. The currents were normalized according to the extrapolated maximum current delivered by the Hill adjustment. In this way, the concentration that generates 50% of the maximum effect (EC_50_) and the Hill coefficient (n_H_) for each situation were obtained.


$$I=I_{0}+\frac{Imax{\left[Glut\right]}^{nH}}{{{EC}_{50}}^{nH}+{\left[Glut\right]}^{nH}}$$ (1)

The analyses were done using the Excel or Sigmaplot 12.3 programs. Statistical figures are given as averages with their standard deviation. Multiple comparisons were made by ANOVA with Bonferroni correction or by Kruskal-Wallis one way ANOVA on ranks. Comparisons between two samples were done by t-test. One sample t-test was used to ascertain whether effects were significantly greater than zero. Statistical analyses were performed using Sigmaplot 12.3 and P <0.05 was considered as the significant level. The number of experiments quoted (n) refer to the number of independent oocytes assayed. They originate from at least 2 separate oocyte isolation procedures from different frogs.

## Supporting information

S1 Table Primers used in cloning CrGluCl subunits. (DOCX)

S1 Fig Schematic representation of GluCl transcripts from database mining. (TIF)

S2 Fig Comparison of *Caligus rogercresseyi* GluCl subunits with putative *Lepeophtheirus salmonis* GluCls predicted amino acid sequences. (TIF)

S3 Fig Effect of emamectin on the response of CrGluCl-B to glutamate. (TIF)
