# Supplementary material for: A new family of glutamate-gated chloride channels in parasitic sea louse Caligus rogercresseyi: A subunit refractory to activation by ivermectin is dominant in heteromeric assemblies
Source: PLoS Pathog. 2023 Mar 14;19(3):e1011188. doi: 10.1371/journal.ppat.1011188 (PMC10038264; doi:10.1371/journal.ppat.1011188)
Supplement: S1 Table — (DOCX) [file ppat.1011188.s001.docx]

| Group | Primers | | Amplicon |
| --- | --- | --- | --- |
|  | Forward (5'-3') | Reverse (5'-3') |  |
| B | GGATCCtttcataatttgcagccagg | TCTAGAaatgtatgaagattattaatagc | 1538 bp |
| C | GGATCCgggcctgagcgattctgagc | TCTAGAaatatcatccggagggagttcc | 1634 bp |
| D | Agagggtctccggaatcg | agtagttggaggaatgttgttc | 1665 bp |
| E | Atggcgattggaaacatattcg | gtcaaaccatggaaaagagg | 1577 bp |

**Supplementary Table 1**. Primers were designed to encompass the full length open reading frame ORF nucleotides for each of the four B-E subunits, from the initial methionine to the stop codon. Capital letters in Forward and Reverse primers in groups B and C are BamHI and XbaI restriction sites respectively.
