# Supplementary figures and images for: A new family of glutamate-gated chloride channels in parasitic sea louse Caligus rogercresseyi: A subunit refractory to activation by ivermectin is dominant in heteromeric assemblies
Source: PLoS Pathog. 2023 Mar 14;19(3):e1011188. doi: 10.1371/journal.ppat.1011188 (PMC10038264; doi:10.1371/journal.ppat.1011188)

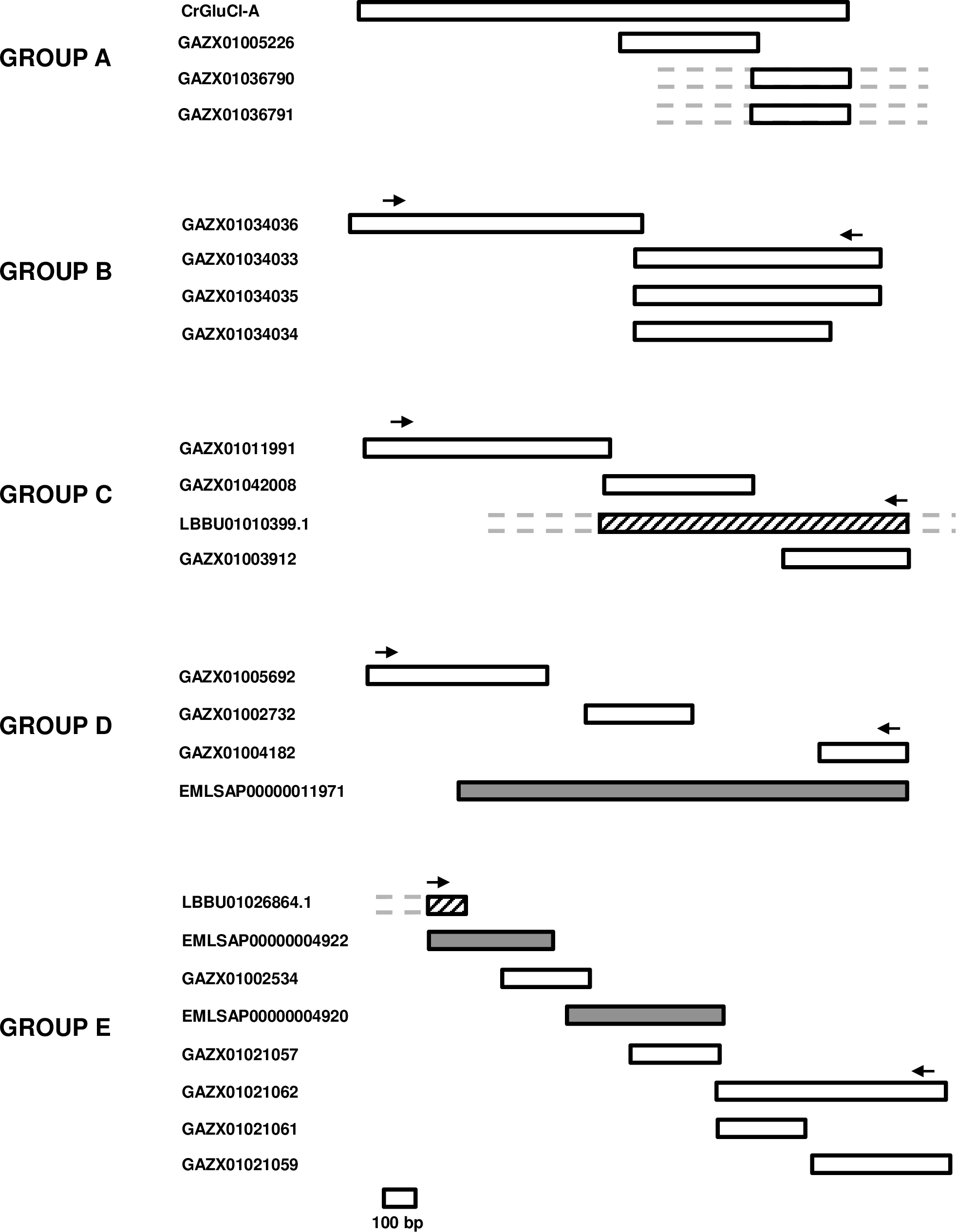

Supplement: S1 Fig — (TIF) [file ppat.1011188.s002.tif]

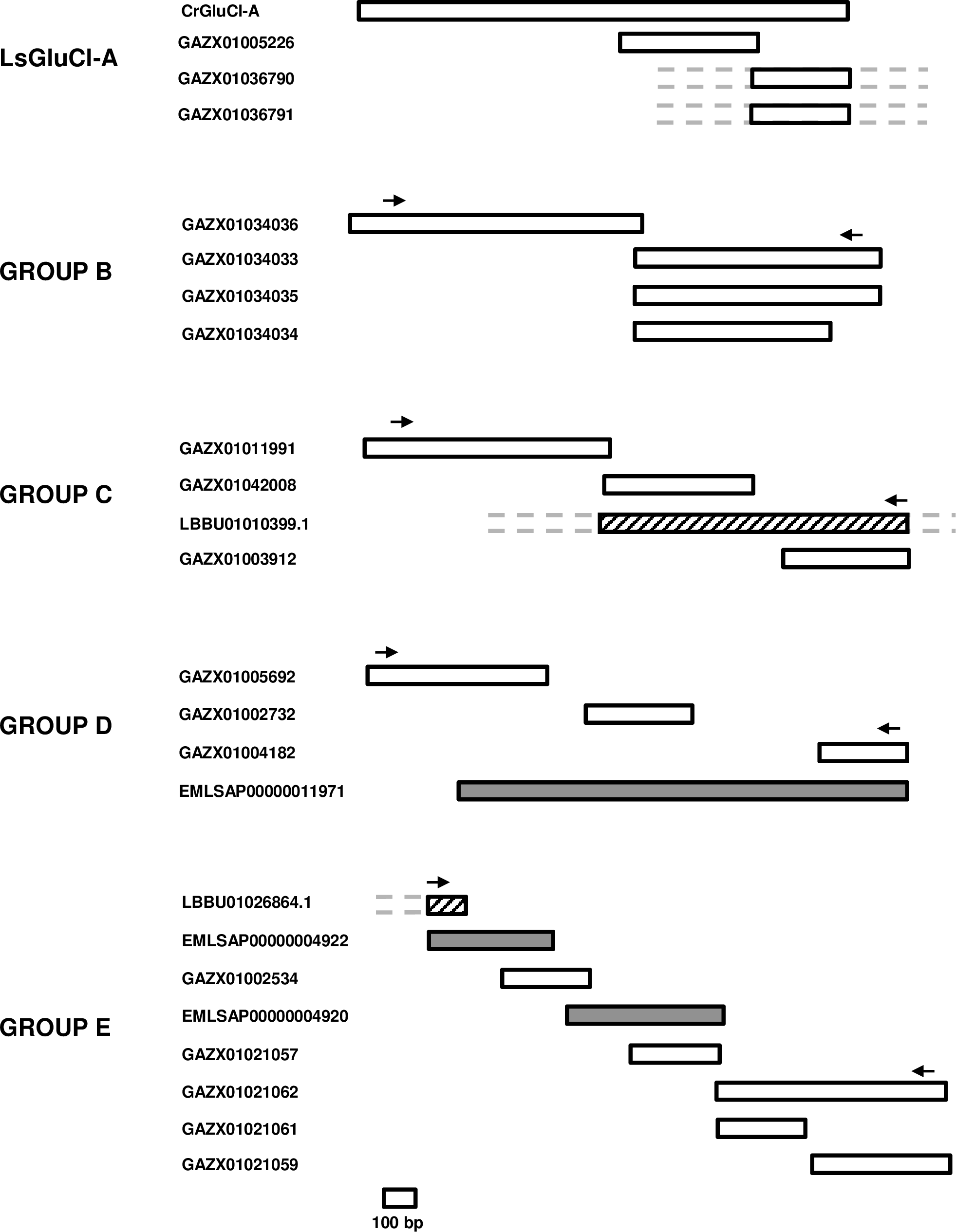

Supplement: S2 Fig — (TIF) [file ppat.1011188.s003.tif]

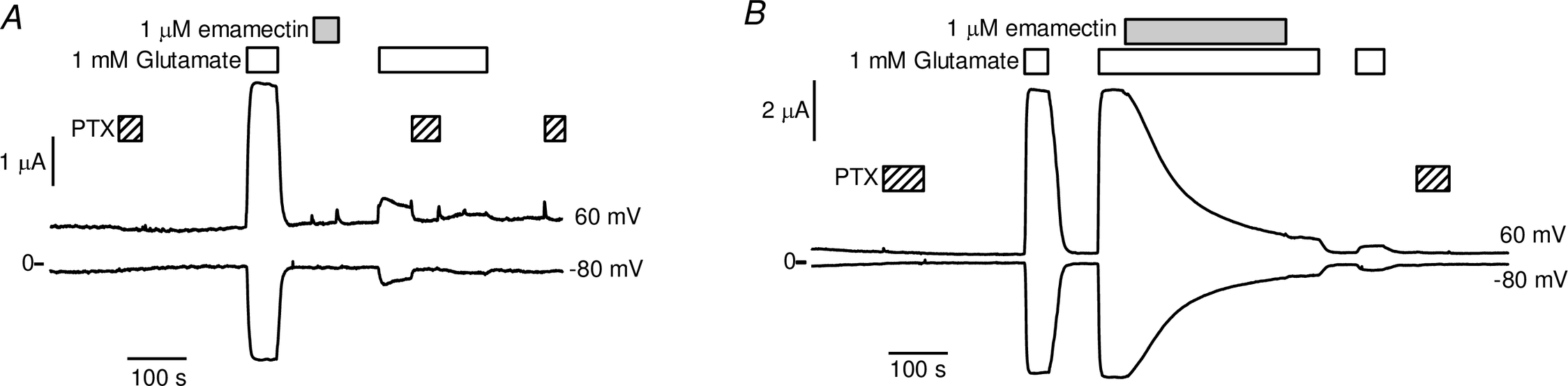

Supplement: S3 Fig — (TIF) [file ppat.1011188.s004.tif]
